# Are behavioural and inflammatory profiles different according to type of stressor, developmental stage, and sex in rodent models of depression? A systematic review

**DOI:** 10.1038/s41380-025-03138-2

**Published:** 2025-08-21

**Authors:** T. M. L. McKenzie, A. Bellato, K. Ismail, C. Fernandes

**Affiliations:** 1 Department of Psychological Medicine, Institute of Psychiatry, Psychology and Neuroscience, London, UK; 2 School of Psychology, University of Southampton, Southampton, UK; 3 Centre for Innovation in Mental Health, University of Southampton, Southampton, UK; 4 Institute for Life Sciences, University of Southampton, Southampton, UK; 5 School of Psychology, University of Nottingham, Semenyih, Malaysia; 6 Mind and Neurodevelopment (MiND) Research Group, University of Nottingham, Semenyih, Malaysia; 7 Social, Genetic and Developmental Psychiatry Centre, Institute of Psychiatry, Psychology and Neuroscience, King’s College London, London, UK; 8 MRC Centre for Neurodevelopmental Disorders, Institute of Psychiatry, Psychology and Neuroscience, King’s College London, London, UK

**Keywords:** Depression, Neuroscience

## Abstract

**Background:**

The onset and persistence of major depressive disorder (MDD) are influenced by various stressors, but the specific impact of different stress types, developmental stages, and sex on behavioural and inflammatory profiles remains unclear.

**Goal and methods:**

We conducted a PRISMA-adhering comprehensive review to systematically examine rodent models of depression and determine how physical, psychological, and physiological stressors - at different developmental stages and across sexes - affect depressive-like behaviours and inflammatory responses.

**Results:**

Utilizing data from Medline, EMBASE, PsycINFO, and Scopus from March 1966 to July 2024, our systematic review of 4886 studies indicate that distinct stressors elicit unique cytokine profiles and behavioural outcomes, with significant variability observed across different developmental stages and between sexes.

**Conclusions:**

The methodological inconsistencies and varying quality of reporting identified by this systematic review highlight the necessity for a consensus for standardized protocols in preclinical studies. Understanding these differences is crucial for developing more effective and personalized strategies for the prevention and treatment of MDD.

## Introduction

Stress is one of the key risk factors for major depressive disorder (MDD) [1, 2], which has a prevalence rate of 5% worldwide [3, 4]. MDD is a highly heterogeneous disorder, affecting people of all ages, genders, socioeconomic status, and ethnicities. Although MDD can emerge at any age, the risk tends to increase during adolescence and early adulthood, and with females being more likely to be diagnosed than males as observed across different cultures and lifespan. Risk factors for MDD include genetic predisposition and stress as a significant environmental factor. Numerous biological systems have been implicated in the pathogenesis of depression, including innate inflammation [5] and dysregulation of the hypothalamic–pituitary-adrenal (HPA) axis [6], which are recognised to be moderated by stress. Despite the key role played by stress in MDD, further research is needed to understand individual differences in response to various stressors across the lifespan and by sex [7, 8]. This potential variation could help classify the complexity and heterogeneity presented in MDD thus supporting more tailored diagnostic algorithms and treatment [9–11]. Analysing the effect of different sources or types of stress in the context of risk of MDD is challenging in clinical cases. On the other hand, it is possible to separate the different types of stress in translatable experimental studies where exposures can be controlled (e.g., in animal studies).

Rodent models are a key tool for studying the aetiology and progress of depression, with one of the most common methods used to induce depressive-like behaviour being subjecting the rodent to stressful stimuli [12]. This is usually accompanied by a marked inflammatory profile characterised by increased levels of certain cytokines, increased microglial activation and an increased accumulation of monocytes and macrophages [12, 13]. Nevertheless, there is conflicting evidence regarding the type of inflammation that is triggered by the stress and significant variation has been observed between different stress exposure models. Some authors have suggested this difference might be attributed to the type of stressor [14]. Despite the lack of a clear classification of stressors, they can be broadly defined as physical, psychological, and physiological/systemic stressors. Physical stressors are those involving physical discomfort or pain with no or limited intentional psychological elements present. Psychological or psychogenic stressors are those involving impairment of some aspect of psychological functioning, and systemic or physiological stressors are those involving cardiovascular, respiratory, immune, and/or metabolic challenges [9, 15]. Psychosocial stressors are typically discussed in human studies but as this includes several societal factors (e.g., economic status, education, culture) which cannot be modelled in rodents. This review only refers to psychological stressors which relate to social interactions/relationships between individuals (family, peer, conspecifics). Although psychological elements may contribute to all stress responses, this review adopts the classification framework proposed by Du Preez et al. [9] which focusses on the main component of the stress manipulation rather than secondary or associated aspects. However, we acknowledge that all physical stressors will have both physical and psychological components and while there are other classification systems available, there is no universally accepted classification [16]. In psychiatric research, validated models exist for physical, psychological and social stressors. As the inflammatory theory of depression has been gaining traction, evidence seems to suggest the type of stressor matters when it comes to inflammatory readouts. For instance, social defeat coupled with LPS injection (psychological and physiological stressors) increased the production of interleukin (IL)-6, tumour necrosis factor-α (TNF-α), and monocyte chemoattractant protein-1 (MCP-1) [17], while inescapable shock (physical stressor) increased IL-1β [18]. On the other hand, unpredictable chronic mild stress, a model of a combination of psychological and physical stress, induced microglial activation but serum levels of cytokines were not affected [19, 20]. Other results are much more robust in showing differences; Du Preez et al. [14] found that while repeated saline injections decreased serum levels of TNF-α, social isolation increased them. In animal studies, developmental stage and sex seem to also play a role as the inflammatory response to social defeat appears to be increased in older mice compared to younger ones [21]. A recent meta-analysis showed that stress introduced during early life in both males and females increases the levels of IL-1β, IL-6, and TNF-α but not IL-10^22^ which contrasts with a decrease in the expression of this anti-inflammatory cytokine is in stressed adult female mice [23].

Different types of stress may be triggering unique changes in biological pathways [15]. There is evidence to support that rodents do respond differently to different types of stress [14, 24–26], it is not possible to know whether animals experience these stressors in the same way as humans, making translation challenging. However, cataloguing the effects of different stressors on a wide range of both physiological and behavioural readouts in both clinical cases and rodent models would at least allow for more relevant comparisons to be made. To the best of our knowledge, the difference in inflammatory responses by type of stressor, developmental stage, and sex in rodent models of depression has not been analysed. The reasons for this gap in knowledge are a) the high variability of animal model studies that use a combination of stressors to induce the depression-like behaviour, b) a lack of consensus or a general classification of cytokines and c) quality of reporting of the specific methodology and readouts of these models.

The present article aims to explore whether behavioural and inflammatory profiles differ according to type of stressor, developmental stage, and sex in rodent models of depression. There is both clinical and preclinical evidence for different stressors have distinct behavioural and/or pathophysiological outcomes. While the effects of different types of stress on behavioural and neurobiological outcomes associated with depression in rodent models has been qualitatively reviewed [9], this review did not focus on specific profiles of key inflammatory markers such as cytokines as these were more simply grouped into anti- or pro-inflammatory cytokines, and only exposure to stress in adult rodent models was considered. The objective of this systematic review was to summarise the patterns of associations between type of stressor and behavioural and inflammatory profiles in rodent models of depression, and to evaluate whether any associations were modified by developmental stage and/or sex.

## Methods

We conducted a systematic review according to the Preferred Reporting Items for Systematic Reviews and Meta-Analyses (PRISMA) guidelines [27] (see Supplementary Appendix 1 for further detail). Details of the protocol, and any protocol amendments, for this systematic review were registered on PROSPERO on 10 January 2023 [28] and can be accessed at https://www.crd.york.ac.uk/prospero/display_record.php?ID=CRD42023384227.

### Search strategy

Medline, EMBASE, PsycINFO and Scopus databases were searched systematically to select studies for review from March 1966 to July 2024. The search strategy included a combination of the following medical subject heading terms and text words: “depression”, “depressive-like-behaviour”, “animal models”, “inflammation”, and “developmental stage”. The search was limited to full text articles and English language studies only. For quality assurance, only articles in peer reviewed journals were included. The complete list of search terms is provided in Supplementary Appendix 1.

Studies were screened in two stages: (i) reviewing all identified titles and abstracts to create a longlist, and (ii) performing a comprehensive full text review on all studies meeting the predefined eligibility criteria. Screening was performed independently by two authors (TMLM and CF). There was a substantial level of reliability of agreement (Cohen’s kappa of 0.63) between the two independent screeners [29] with complete agreement on the final list following a discussion of the discrepancies in the shortlist. Data extracted from the final shortlisted articles included: first author, year, title, main objective of the study, species, strain, sex, developmental stage, stressor, classification of stressor (psychological, physiological, or physical; and combined if a mix of stressors was used), housing condition, life stage when exposed, depressive measures, inflammatory markers (the mean, the standard deviation (SD), and the number of animals studied) and assay method, metabolic profile, hormonal measures, neurotransmitter measures, cellular changes, and ethics. In case of missing data, the corresponding author of the article was contacted by email and one reminder sent in case of no answer to the initial email. The complete data extraction sheet is presented in the Supplementary Material.

Data synthesis included a narrative review. Given that our main outcome (cytokines) is a quantitative continuous variable, standardised mean differences and 95% confidence intervals for cytokines levels between non-stressed control animals and stressed animals were calculated. All results are presented by type of stressor, sex, and developmental stage.

### Selection criteria

**Fig. 1:**
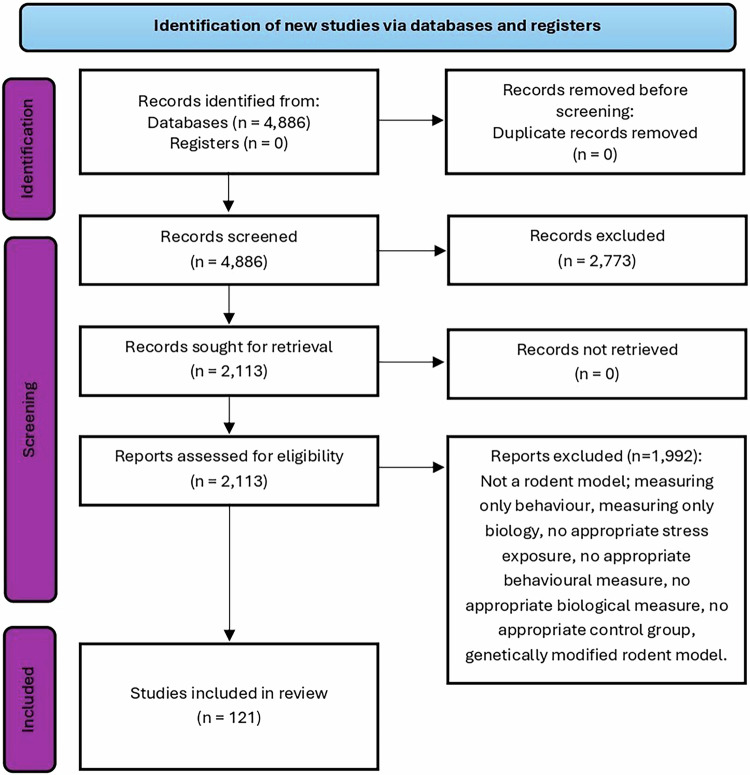
PRISMA flow diagram depicting the search strategy employed, the number of studies identified, excluded, and included at each stage of the review. The number of studies exceeds the number of publications included in the review as several studies include multiple outcomes, such as investigations employing various versions of stress exposure but conducted within the same publication.

A total of 121 studies were included in this systematic review, as shown in Fig. 1. Only in-vivo studies on rodents (mice or rats) aimed at modelling depression (depressive-like behaviour) with a separate, non-stressed control group were included. Given that depression-like behavior in rodents is often assessed using multiple validated measures per study, a study was included if experimental animals exhibited depressive-like behavior in at least one validated measure compared to controls. Studies where the control group involved vehicle injections and/or sham surgery were excluded as these procedures could result in some level of stress exposure. We included all preclinical animal studies using mouse and rat models of stress conducted in male and/or females, where stress exposures occurred at any/all stages of development (prenatal, early postnatal, adolescence and adulthood). Early postnatal was defined as between postnatal day (PND) 0 and 21, adolescence between PND21 and 60, and adulthood in animals greater than eight weeks old [30, 31]. For articles which did not state the age of the animals, this was estimated using growth charts available from a range of commercial suppliers (see Supplementary Appendix 1). Although other rodent species have been used in this area of research, mice and rats are the most widely used, so this review only included articles from these rodent species [32]. In addition, only studies that measured inflammation and gave a proper account of the assay used, provided sufficient information to describe the type of stressor used and behavioural measurements were included. Many studies investigating cellular inflammatory responses, such as peripheral white blood cells and glial cell activation, were captured in the first search strategy. However, these studies subsequently were not included as these studies lacked appropriate controls or depression-like behavioural changes, resulting in a primary focus on stress-induced changes in cytokine levels. Studies where the cytokines were not measured in brain, serum, plasma or cerebrospinal fluid were excluded as the review is looking to associate cytokine profiles to brain-mediated effects such as behaviour. In addition, genetic rodent models of depression were excluded because they potentially include a multiple hit strategy of combining genetic factors with stress exposure and would be difficult to compare to studies in more genetically homogeneous animals. Studies where the immune system was experimentally challenged at the end of a stress protocol were not included. Given the inclusion criteria require both changes in inflammatory responses and changes in depression-like behaviours, studies where these changes were not associated were excluded which leads to a bias towards inclusion of positive findings in depression models. Therefore, this review cannot comment on the robustness/reproducibility of specific depression models. A summary of the studies included in this review, including the stress types, species, strains, sex, and life stages of the experimental rodents, is presented in Supplementary Table 1.

**Fig. 2:**
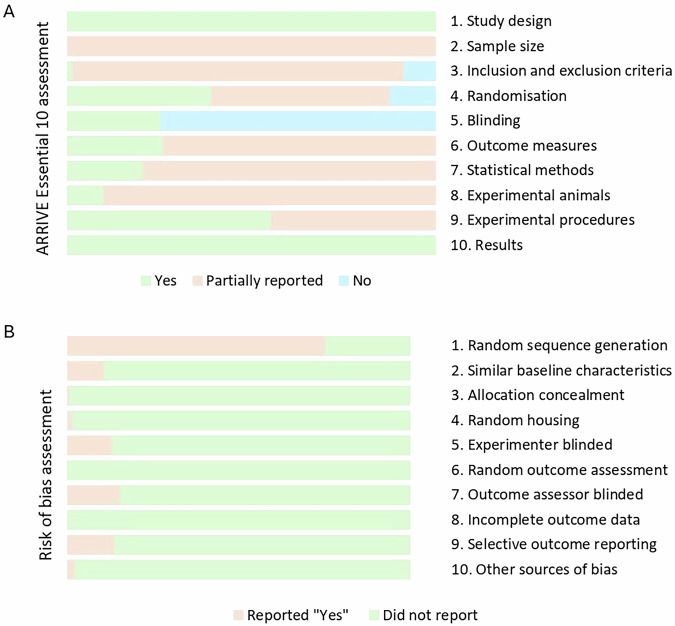
Methodological consistency and quality of the articles reviewed. **A**. Cumulative bar charts showing the reliability assessment of findings across 10 items outlined in the ARRIVE (Animal Research: Reporting of In Vivo Experiments) Essential 10 guidelines. **B**. Cumulative bar charts showing SYRCLE’s risk of bias assessment.

To ensure methodological consistency and quality, we used the ARRIVE guidelines (Animal Research: Reporting of In Vivo Experiments) checklist of information to include in publications describing animal research [33] (Fig. 2A and Supplemental ARRIVE guidelines checklist) and the SYRCLE’s risk of bias tool for animal studies (Fig. 2B) [34] were used. Given the ethical concerns of early studies, our quality assessment also included an ethical appraisal to discuss the reproducibility of the methods in current research.

### Stress exposure models

Stress models employing physiological, psychological, and/or physical stimuli aimed to induce depression-like behaviours were included in the review. Previous reviews have attempted to classify these models into physical (e.g., injection stress, restraint stress, temperature stress), psychological (e.g., social isolation, exposure to dominant conspecifics, predator stress), or combined (physical and psychological) models [9]. However, this may result in some classification bias so for this review, initial comparisons were made based on the stress model itself without classification before secondary comparisons were made using the classifications proposed by Du Preez et al. [9]. In addition to stress-classification, consideration was also given to the severity of the stress exposure used, and studies ranked according to the number and duration of stress bouts.

### Outcome measures

The main outcome measures extracted from the studies were the inflammatory profiles of cytokines. More than 30 cytokines (inflammatory profiles) have been described in the literature and several types of classification exist and are used across the field. For this review, all cytokines were considered relevant for comparison and classified into TH1 (IL-2, IL-12, IFN-β, IFN-γ) versus TH2 (IL-4, IL-5, IL-13) cytokines, and pro-inflammatory (CCL, CXCL, IL-1, IL-6, IL-8, IL-15, IL-17, IL-18, IL-21, IL-22, IL-24, IL-33, IFN-α, MCP-1, TNF-α) versus anti-inflammatory (IL-1ra, IL-9, IL-10, IL-24, IL-37, TGF-β) cytokines; classifications based on Himmerich et al. [35].

To comprehensively assess the impact of different stressors in rodent models of depression, secondary outcome measures included behavioural readouts [36], stress-related hormones, key brain metabolites and cellular phenotypes previously reported association with depression [9, 37].

## Results

### Impact of species type on stress outcomes

In assessing the impacts of exposure to various stress types, rodents were initially categorized by species type. Among the 130 studies (from 121 publications) included in this systematic review, 62% (*n* = 80) employed mice, including C57BL/6 (*n* = 61), ICR (*n* = 7), BALB/c (*n* = 8), Kunming (KM) outbred (*n* = 3), and Swiss (*n* = 2) strains. In contrast, 38% (*n* = 50) utilized rats, with 33 studies employing Sprague Dawley and 17 studies using the Wistar strain of rats. As illustrated in Supplementary Table 2, few differences were observed between mice and rats. To scrutinize the primary outcomes relevant to this systematic review, we methodically evaluated cytokines with protein-level data documented in no fewer than ten studies (*n* ≥ 10). This conservative approach enabled a thorough comparison of the protein levels of IL-1β, IL-6, TNF-α, and IL-10 between the two species types under investigation. Interestingly, all four cytokines exhibited minimal differences between mice and rats, indicating consistent stress response patterns across species type. However, it is noteworthy that the studies involving mice often reported multiple measurements for the same cytokine. This is due to the use of various tissue types for sampling purposes. Conversely, rat studies tended to sample from a single tissue type, resulting in a reduced number of cytokine measurements reported compared to studies involving mice.

This review focused on primary outcome measures; however, secondary outcomes (including behavioural, hormonal, and cellular changes) were also documented in Supplementary Appendix 2. Secondary outcome trends further supported the similarity of stress-induced effects across both species type, with indications of depression-like and anxiety-like behaviours observed, along with elevated corticosterone levels and increased Iba-1 expression, indicating microglial activation. Given the limited species differences observed, the results from mouse and rat studies subsequently were combined in our review.

### Impact of sex on stress outcomes

Next, an assessment of the influence of sex on stress outcomes was conducted, presented alongside the species type comparison in Supplementary Table 2. The analysis revealed that 86% (*n* = 112) of the studies utilized male animals, while 9% (*n* = 12) either exclusively used female subjects or included both sexes but analysed them separately. Notably, only 3% (*n* = 4) of studies combined data from both sexes into a single result, and 2% (*n* = 3) did not specify the sex of the rodents. Due to the limited number of studies involving female rodents, a comprehensive comparison between sexes was not possible. However, significant similarities across male and female rodents were observed, particularly in the post-stress elevation of pro-inflammatory cytokine levels as well as anhedonia-like behaviours, decreased serotonin levels, and increased expression of microglial markers. Given these key similarities and lack of studies in female rodents, data from both sexes were combined for subsequent syntheses.

### Impact of life stage on stress outcomes

Building on the analysis of species type and sex differences, an examination of the impact of life stage on rodent models of depression was undertaken. We discovered that stress induction occurred in adulthood in 45% (*n* = 58) of the studies and in adolescence in 46% (*n* = 60) of the studies. The remainder of studies comprised the early postnatal period (7%, *n* = 9) and the prenatal life stage (2%, *n* = 3). Given the substantial prevalence of stress induction across adult and adolescent life stages of rodents within the literature, our review emphasized these two developmental periods. This review focused on the primary outcomes of studies conducted during adulthood and adolescence, categorized by stress type, outlined in Table 1 and Table 2, respectively. A comprehensive examination of secondary outcomes within adulthood versus adolescent exposure cohorts was also undertaken, see Supplementary Table 3 and Supplementary Table 4, respectively.

**Table 1:** Breakdown of primary outcome phenotypes associated with stress exposure in adult rodents (*n* = 58).

Outcome measure	Total number of studies measuring the outcome of interest (% total studies with specific outcome of significant increase ▴ or decrease ▾)	Number of studies with specific outcome of ▴Significantly increased^a^ ▾Significantly decreased^a^ - No significant difference^a^														
		UCMS/CMS/ CVS (*n* = 33)			Restraint stress (*n* = 8)			CSDS/SDS/RSDS (*n* = 8)			Maternal stress (*n* = 1)			Other types of stress (*n* = 8)		
		▴	▾	-	▴	▾	-	▴	▾	-	▴	▾	-	▴	▾	-
Pro-inflammatory cytokines – protein levels																
IL-1α	1 (100%▴)	1	0	0	0	0	0	0	0	0	0	0	0	0	0	0
IL-1β	58 (84%▴)	30	0	1	5	0	3	7	1	2	1	1	1	6	0	0
Pro-IL-1β	1 (100%▴)	1	0	0	0	0	0	0	0	0	0	0	0	0	0	0
IL-6	39 (77%▴)	13	2	2	1	0	0	7	0	3	2	0	1	5	0	0
IL-17	2 (50%▴)	1	0	1	0	0	0	0	0	0	0	0	0	0	0	0
IL-17A	1 (0%▴)	0	1	0	0	0	0	0	0	0	0	0	0	0	0	0
IL-17F	1 (0%▴)	0	1	0	0	0	0	0	0	0	0	0	0	0	0	0
IL-18	8 (100%▴)	0	0	0	2	0	0	6	0	0	0	0	0	0	0	0
CXCL1	6 (100%▴)	0	0	0	0	0	0	6	0	0	0	0	0	0	0	0
MCP-1	3 (67%▴)	2	0	1	0	0	0	0	0	0	0	0	0	0	0	0
TNF-α	48 (88%▴)	24	0	3	3	0	1	9	0	0	1	0	2	5	0	0
Pro-inflammatory cytokines – RNA levels																
IL-1α	1 (100%▴)	1	0	0	0	0	0	0	0	0	0	0	0	0	0	0
IL-1β	35 (66%▴)	15	0	5	2	0	0	1	1	5	0	0	0	5	0	1
IL-1ra	2 (0%▴)	0	1	1	0	0	0	0	0	0	0	0	0	0	0	0
IL-6	21 (48%▴)	8	0	2	0	0	0	0	0	6	0	0	0	2	0	3
IL-8	1 (0%▴)	0	0	0	0	0	0	0	0	0	0	0	0	0	0	1
IL-15	1 (0%▴)	0	0	0	0	0	0	0	0	0	0	0	0	0	0	1
IL-17	2 (50%▴)	1	0	1	0	0	0	0	0	0	0	0	0	0	0	0
IL-18	1 (100%▴)	0	0	0	1	0	0	0	0	0	0	0	0	0	0	0
CXCL10	1 (100%▴)	0	0	0	0	0	0	1	0	0	0	0	0	0	0	0
TNF-α	33 (55%▴)	13	0	7	0	0	0	0	0	6	0	0	0	5	0	2
Anti-inflammatory cytokines – protein levels																
IL-10	16 (56%▾)	2	6	3	0	1	0	0	0	1	0	2	1	0	0	0
TGF-β	6 (17%▾)	3	1	2	0	0	0	0	0	0	0	0	0	0	0	0
Anti-inflammatory cytokines – RNA levels																
IL-10	7 (29%▾)	2	2	3	0	0	0	0	0	0	0	0	0	0	0	0
TGF-β	3 (100%▾)	0	3	0	0	0	0	0	0	0	0	0	0	0	0	0
IL-1ra	2 (50%▾)	0	1	1	0	0	0	0	0	0	0	0	0	0	0	0
TH1 cytokines – protein levels																
IL-2	1 (100%▴)	1	0	0	0	0	0	0	0	0	0	0	0	0	0	0
IFNy	5 (40%▴)	2	0	3	0	0	0	0	0	0	0	0	0	0	0	0
TH1 cytokines – RNA levels																
IL-2	1 (0%▴)	0	0	0	0	0	0	0	0	0	0	0	0	0	0	1
TH2 cytokines – protein levels																
IL-4	7 (29%▾)	2	0	3	0	2	0	0	0	0	0	0	0	0	0	0
TH2 cytokines – RNA levels																
IL-4	5 (40%▾)	0	1	2	0	0	0	0	0	0	0	0	0	0	1	1

The number of studies exceeds the number of publications included in the review as several studies include multiple outcomes, such as investigations employing various versions of stress exposure but conducted within the same publication. Maternal stress includes maternal care deprivation (*n* = 1). Other stress types include forced swim stress (*n* = 2), ultrasound stress (*n* = 1), sleep deprivation (*n* = 2), and injection stress (*n* = 3).

Stress types: *CDS* chronic defeat stress, *CMS* chronic mild stress, *CSDS* chronic social defeat stress, *CVS* chronic variable stress, *RSDS* repeated social defeat stress, *SDS* social defeat stress, *UCMS* unpredictable chronic mild stress. Biological: *CXCL* CXC chemokine ligand, *IFN* interferon, *IL* interleukin, *MCP* monocyte chemoattractant protein-1, *RNA* ribonucleic acid, *TGF* transforming growth factor, *TH* T helper, *TNF* tumour necrosis factor.

^a^Relative to stress-free control rodents.

**Table 2:** Breakdown of primary outcome phenotypes associated with stress exposure in adolescent rodents (*n* = 60).

Outcome measure	Total number of studies measuring the outcome of interest (% total studies with specific outcome of significant increase ▴ or decrease ▾)	Number of studies with specific outcome of ▴Significantly increased^a^ ▾Significantly decreased^a^ - No significant difference^a^											
		UCMS/CMS/ CVS (*n* = 47)			Restraint Stress (*n* = 7)			CSDS/SDS/RSDS (*n* = 4)			Maternal Stress (*n* = 2)		
		▴	▾	-	▴	▾	-	▴	▾	-	▴	▾	-
Pro-inflammatory cytokines – protein levels													
IL-1β	48 (▴98%)	42	0	1	3	0	0	0	0	0	2	0	0
IL-1β (cleaved)	1 (▴100%)	1	0	0	0	0	0	0	0	0	0	0	0
IL-6	45 (▴93%)	33	0	1	6	0	1	4	0	2	1	0	0
IL-8	1 (▴100%)	1	0	0	0	0	0	0	0	0	0	0	0
IL-17A	1 (▴100%)	1	0	0	0	0	0	0	0	0	0	0	0
IL-18	8 (▴100%)	8	0	0	0	0	0	0	0	0	0	0	0
IL-33	1 (▴0%)	0	1	0	0	0	0	0	0	0	0	0	0
MCP-1	2 (▴100%)	2	0	0	0	0	0	0	0	0	0	0	0
TNF-α	49 (▴98%)	39	0	0	6	0	1	1	0	0	2	0	0
Pro-inflammatory cytokines – RNA levels													
IL-1β	18 (▴100%)	17	0	0	0	0	0	0	0	0	1	0	0
IL-6	12 (▴100%)	10	0	0	1	0	0	1	0	0	0	0	0
IL-18	5 (▴100%)	5	0	0	0	0	0	0	0	0	0	0	0
CCL-1	3 (▴100%)	3	0	0	0	0	0	0	0	0	0	0	0
CCL-2	1 (▴100%)	1	0	0	0	0	0	0	0	0	0	0	0
CXCL-1	3 (▴100%)	3	0	0	0	0	0	0	0	0	0	0	0
TNF-α	20 (▴95%)	16	0	0	1	0	0	1	0	1	1	0	0
Anti-inflammatory cytokines – protein levels													
IL-1ra	1 (▾100%)	0	1	0	0	0	0	0	0	0	0	0	0
IL-9	1 (▾0%)	0	0	1	0	0	0	0	0	0	0	0	0
IL-10	7 (▾100%)	0	6	0	0	1	0	0	0	0	0	0	0
IL-24	1 (▾0%)	1	0	0	0	0	0	0	0	0	0	0	0
IL-37	1 (▾0%)	1	0	0	0	0	0	0	0	0	0	0	0
TGF-β	2 (▾100%)	0	2	0	0	0	0	0	0	0	0	0	0
Anti-inflammatory cytokines – RNA levels													
IL-10	6 (▾67%)	1	4	1	0	0	0	0	0	0	0	0	0
TGF-β	4 (▾50%)	2	2	0	0	0	0	0	0	0	0	0	0
Cytokines that have both pro-inflammatory and anti-inflammatory properties – protein levels													
CX3CL1	2 (▴0%)	0	0	0	0	0	0	0	2	0	0	0	0
CXCL12	1 (▴100%)	1	0	0	0	0	0	0	0	0	0	0	0
TH1 cytokines – protein levels													
IFN-β	1 (▴100%)	1	0	0	0	0	0	0	0	0	0	0	0
IFNγ	2 (▴50%)	1	0	0	0	0	1	0	0	0	0	0	0
TH1 cytokines – RNA levels													
IFNγ	1 (▴100%)	1	0	0	0	0	0	0	0	0	0	0	0
TH2 cytokines – protein levels													
IL-4	2 (▾50%)	1	1	0	0	0	0	0	0	0	0	0	0
TH2 cytokines – RNA levels													
IL-4	5 (▾20%)	4	1	0	0	0	0	0	0	0	0	0	0

The number of studies exceeds the number of publications included in the review as several studies include multiple outcomes, such as investigations employing various versions of stress exposure but conducted within the same publication. Maternal stress includes maternal care deprivation (*n* = 1) and maternal sleep deprivation stress (*n* = 1).

Stress types: *CDS* chronic defeat stress, *CMS* chronic mild stress, *CSDS* chronic social defeat stress, *CVS* chronic variable stress, *RSDS* repeated social defeat stress, *SDS* social defeat stress, *UCMS* unpredictable chronic mild stress. Biological: *CCL* chemokine ligand, *CXCL* CXC chemokine ligand, *IFN* interferon, *IL* interleukin, *MCP* monocyte chemoattractant protein, *RNA* ribonucleic acid, *TNF* tumour necrosis factor, *TGF* transforming growth factor, *TH* T helper.

^a^Relative to stress-free control rodents.

### Stress and the associated primary outcome measures in rodents exposed to stressors during adulthood (Table 1)

Within the category of stress induction beginning during adulthood, unpredictable chronic mild stress (UCMS) was the most common stress procedure utilized, accounting for 56.0% (*n* = 33) of studies. The remaining stress categories included restraint stress (13.5%, *n* = 8), CSDS (15.3%, *n* = 9), maternal stress (1.7%, *n* = 1), and other stressors (13.5%, *n* = 8). Across these stress paradigms, IL-1β, IL-6, and TNF-α were the most frequently observed pro-inflammatory cytokines. At the protein level, significant elevations were noted for IL-1β in 84% (*n* = 49) of studies, IL-6 in 77% (*n* = 30), and TNF-α in 88% (*n* = 42). Importantly, non-significant changes in pro-inflammatory protein levels, leading to less than 100% increase post-stress, primarily arose from stress types other than UCMS. For instance, studies on restraint stress reported insignificant changes in 38% (*n* = 3) of cases, while UCMS studies indicated increases across 98% (*n* = 30) of instances. Although RNA levels were less commonly assessed, they were reported in a subset of studies and compared across stress types when data were available from at least five studies. The elevation of pro-inflammatory cytokine RNA levels post-stress was less pronounced than the corresponding protein increases, consistent with the understanding that RNA and protein levels do not always correlate [38]. Nonetheless, significant increases in RNA levels were observed for IL-1β in 66% (*n* = 23) of studies, for IL-6 in 48% (*n* = 10) of studies, and for TNF-α in 55% (*n* = 18) of studies.

The outcomes of anti-inflammatory cytokines following stress during adulthood displayed greater variability, which may be attributed to their less frequent measurement across the reviewed studies. Notably, significant reductions in IL-10 were observed in 56% (*n* = 9) of studies at the protein level and in 29% (*n* = 2) at the RNA level. A comprehensive evaluation of TH1 and TH2 cytokines was challenging due to the limited number of studies. Among TH2 cytokines, IL-4 was the most frequently reported, with decreased protein levels observed in 29% (*n* = 7) of studies and reduced RNA levels reported in 40% (*n* = 5) of studies.

### Stress and the associated primary outcome measures in rodents exposed to stressors in adolescence (Table 2)

In contrast to the more heterogeneous cytokine profiles observed in rodents exposed to stressors in adulthood, our investigation found a more robust and distinct pattern in rodents exposed to stressors during adolescence; characterized by a consistent elevation in pro-inflammatory cytokines alongside a concurrent decrease in anti-inflammatory cytokines. Additionally, there were far fewer non-significant results reported when stress induction began during the adolescent life stage. As shown in Table 2, across all adolescent rodent studies included in this review, there was a significant increase in pro-inflammatory cytokines IL-1β, IL-6, and TNF-α observed in 98%, 93%, and 98% of cases, respectively. This profile is particularly prominent among studies employing UCMS, the most used stress induction paradigm in our review. In the UCMS category, there was a significant increase observed in 98% (*n* = 42) of cases for IL-1β, 97% (*n* = 33) of measurements for IL-6 and 100% (*n* = 39) of measurements for TNF-α. Notably, RNA levels of various pro-inflammatory cytokines were also increased in most studies that administered stress during adolescence. For instance, RNA levels of IL-1β (*n* = 18), IL-6 (*n* = 12), and IL-18 (*n* = 5) increased across all studies, and TNF-α (*n* = 20) increased significantly in 95% of cases.

Anti-inflammatory cytokine levels were generally reduced following stress exposure during adolescence, with significant decreases observed across all studies measuring protein levels of IL-10 (*n* = 7). Similar to studies investigating stress during adulthood, research examining stress exposure during adolescence provided limited focus on TH1 and TH2 cytokines, so much so that TH1 cytokines were not reported in the minimum number of studies required for comparison in this review. TH2 cytokines were represented by measurements of IL-4, with protein levels assessed in two studies and RNA levels assessed in five studies. Decreases in IL-4 protein levels were observed in 50% (*n* = 1) of cases, while RNA levels were reduced in only 20% (*n* = 1) of cases. Given the small sample size, further studies are needed to draw more robust conclusions regarding these findings.

### Stress and the associated secondary outcome measures in adult and adolescent rodents

In addition to primary outcomes, a supplementary analysis explored secondary outcome measures including behavioural, hormonal, metabolic, neurotransmitter, and neurobiological responses across adulthood and adolescence, outlined in Supplementary Tables 3 and 4, respectively. A key inclusion criterion was the presence of depressive-like behaviour in stressed rodents compared to controls, assessed using established behavioural measures. For instance, reduced sucrose intake was reported in 97% (*n* = 37) of adult studies and in 98% (*n* = 44) of adolescent studies. However, anxiety-like behaviours were less frequently observed, with only 78% (*n* = 25) of both adult and adolescent rodents displaying anxiety-like responses in the open-field test (OFT). The review also identified a relatively consistent increase in corticosterone levels, reported in 78% of adult studies and 88% of adolescent studies, indicating potential dysregulation of the HPA axis in depressive-like states. Markers of oxidative stress, such as elevated malondialdehyde (MDA) levels, and disruptions in the serotonin (5-HT) system, which plays a critical role in mood regulation and stress adaptation, were also observed. However, the impact on 5-HT levels varied, with significant decreases reported in 64% of adult studies and 86% of adolescent studies. The influence of life stage on 5-HT regulation remains unclear due to the reduced number of studies in adults and variations in stress paradigms across age groups. One secondary measure demonstrating even greater variability with its responses was glial fibrillary acidic protein (GFAP), a marker of astrocyte activation. Reductions in GFAP were observed in only 45% of adult studies and 43% of adolescent studies, suggesting inconsistent glial responses to stress across developmental stages.

### Stress and the associated outcome measures in rodents exposed to stress during early developmental stages

Due to the limited number of studies, the effects of stress exposure during early developmental stages were not extensively reviewed. However, we observed that elevated protein levels of pro-inflammatory cytokines were reported in rodents exposed to stress during the early postnatal period (*n* = 9), a stage analogous to childhood in humans, though these elevations were less frequent compared to stress exposure in the prenatal period (*n* = 3; see Supplementary Table 5 and Supplementary Table 6). Variability in cytokine responses was largely associated with stress paradigms other than UCMS. For instance, maternal care deprivation solely resulted in non-significant pro-inflammatory cytokine changes and often failed to produce significant behavioural alterations in early postnatal rodents. In contrast, prenatal exposure to maternal UCMS, light exposure, and restraint stress consistently led to increases in pro-inflammatory cytokine protein levels, along with the emergence of depressive-like behaviours.

## Discussion

The main objective of this systematic review was to comprehensively examine relevant literature to investigate potential variations in inflammatory profiles concerning the type of stress, developmental stage, and sex within mice and rat models of depression. Subsequently, an additional analysis focused on secondary outcome measures including behavioural outcomes, hormonal alterations, and cellular changes associated with stress-induced models of depression as detailed in the Supplementary Material.

In rodent depression models, changes in inflammatory marker profiles are often attributed to cytokine alterations induced by stressful stimuli. Typically, stress induction leads to an upregulation of pro-inflammatory cytokines, such as IL-1β, IL-6, and TNF-α, within specific brain regions associated with mood regulation [39]. Concurrently, there may be a downregulation of anti-inflammatory cytokines, such as IL-10, contributing to an imbalance between pro- and anti-inflammatory signalling [39]. This dysregulated cytokine profile can induce microglial activation and astrocyte reactivity, amplifying neuroinflammatory responses and potentially exacerbating depressive-like behaviours [40]. Additionally, prolonged exposure to stress may further disrupt cytokine balance, perpetuating a chronic inflammatory state implicated in the pathophysiology of depression in rodent models [41].

### The role of life stage in rodent models of depression

Based on the results of the reviewed literature, the developmental stage of rodents significantly influences the impact of stress on both inflammatory and behavioural outcomes. Adult rodents exposed to stress showed varied cytokine profiles, however, the results were leaning toward a trend of increased levels post-stress. It is essential to note that our findings predominantly stemmed from UCMS studies. Rodents subjected to this paradigm exhibited cytokine alterations largely following a pattern of significant increase post-stress, however, they did occasionally deviate from this expected pattern; often manifesting as non-significant outcomes or, less frequently, as significant changes occurring in the opposite direction. For instance, adult studies using the UCMS paradigm revealed mixed results for IL-6 levels, reporting two instances of non-significance and two instances of decreased levels. Additionally, TNF-α demonstrated non-significant results in three adult UCMS studies. Similar trends were observed in studies utilizing less frequently employed stress procedures (e.g., restraint stress, CSDS, maternal stress, forced swim stress, ultrasound stress, and injection stress), indicating that this variability warrants further examination.

As previously mentioned, early life stress has been linked to elevated levels of pro-inflammatory cytokines such as IL-1β, IL-6, and TNF-α [22], which we observed in studies incorporating adolescent rodents. Specifically, adolescent rodents consistently exhibited increased protein levels of pro-inflammatory cytokines and reduced anti-inflammatory cytokines. This suggests that adolescence may be a period of heightened vulnerability to stress-induced inflammation. In considering why adolescent-aged rodents displayed more consistent cytokine trends, one possible reason could be the heightened vulnerability of the adolescent brain to inflammatory processes. Adolescence is characterized by dynamic changes in both brain structure and function, including synaptic pruning, myelination, and neuroplasticity, which render the brain more susceptible to environmental influences, including stress [42, 43]. Consequently, stressors may elicit more pronounced and detectable changes in cytokine levels in adolescent rodents compared to adults, resulting in a lower likelihood of non-significant outcomes. Furthermore, the stress-response systems may play a role in the more synchronized cytokine response found in adolescents. For instance, the HPA axis may be more responsive during adolescence, resulting in increased physiological responses to stressors [42].

This review also considered secondary outcomes encompassing behavioural responses, hormonal responses, and neurobiological indices, spanning various developmental stages. Both adult and adolescent rodents exhibited depression-like behaviours, such as reduced sucrose intake and increased immobility in the FST and/or TST. However, adolescents showed greater variability in behavioural responses, with more frequent non-significant differences compared to controls. This contrasts with the consistent cytokine response observed in adolescents. This variability might be due to the less developed coping mechanisms in adolescents and ongoing maturation of the prefrontal cortex, which continues to develop throughout adolescence, unlike in fully matured adult rodents, which might result in greater variability seen in their behaviour [44, 45]. Additionally, it is important to consider that many rodent behavioral tests were originally designed and validated using adult rodents, who may exhibit different behavioral patterns compared to their adolescent counterparts and therefore may not be sensitive enough to measure changes in adolescents [46, 47]. Regarding stress-related hormones, the predominant increase in corticosterone levels observed across studies, more so in adolescents than adults, underscores the potential involvement of the HPA axis dysregulation in depressive-like states among rodents. Additionally, elevated malondialdehyde levels, a marker of oxidative stress, and disruptions in the serotonin (5-HT) system, which regulates mood and stress adaptation, were also observed. Chronic stress paradigms, such as CSDS, have been shown to disrupt these systems [16]. However, the effect on 5-HT levels varied: 64% of studies in adult rodents and 86% of studies in adolescents reported significant decreases. However, determining the impact of life stage on 5-HT measures is challenging within the context of this review due to the lower number of adult studies that reported 5-HT findings.

Furthermore, the heightened cytokine responses observed during the prenatal period may be attributed to the increased vulnerability of this developmental stage, potentially leading to lasting changes in gene expression [48]. However, it is challenging to distinguish the impact of life stage early in development due to the variability in stressors used in the few studies that examined stress induction in the prenatal and early postnatal periods.

### The role of sex in rodent models of depression

To understand the impact of sex on stress responses, we intended to evaluate sex differences in our outcome measures. However, this analysis was limited by the small number of studies involving female rodents, which constituted only 9% (*n* = 12) of the reviewed studies. Preliminary analysis of these studies indicated greater variability in pro-inflammatory and anti-inflammatory cytokine levels in female rodents compared to males exposed to stressors (Supplementary Table 2). This variability may be due to hormonal fluctuations during the oestrous cycle, which can influence cytokine levels independently of stress exposure [49]. Alternatively, the observed variability might result from sampling bias, as the majority of studies use male rodents. Literature reviews suggest that females generally do not show greater variability across outcome measures compared to males [50–52]. Given the different manifestations of depression in humans by sex [53], it is crucial to include both sexes in depression research to better understand stress as a risk factor. In addition to the lack of studies utilising both sexes, it was particularly concerning that we found four studies in which sex was never stated in the article (Supplementary Table 7). This is just one of the several serious failings in the standards of reporting in the literature we uncovered during the review.

### The role of stress type in rodent models of depression

Finally, our investigation concentrated on whether different stressors and stress types elicited discrete effects on our primary and secondary outcome measures in rodent models of depression. Understanding the effects of different stress paradigms is essential for accurate modelling of depression and the assessment of the specific underlying biological mechanisms triggered by stress in relation to depression.

Among the stress types reviewed, UCMS emerged as the most prevalent and consistently reported stressor, exhibiting notable impacts on inflammatory profiles, behaviour, and other secondary outcome measures. Unlike other stressors, UCMS is characterized by its inherent unpredictability, owing to the continuous and randomized cycling of stressors within the procedure. This lack of a fixed schedule for stress induction aims to minimize adaptation to the stressors encountered and maximise the impact of stress on the rodents exposed to this paradigm. Additionally, UCMS can be classified as both a physical stressor, due to its utilization of stressors such as restraint or forced swim, as well as psychological, due to its typical inclusion of stressors such as overnight illumination and/or predator sounds or smells. The potentially greater impact of the unpredictable nature, the combination of stress types, coupled with larger sample sizes often employed in UCMS studies, may contribute to the significant and consistent trends observed in research utilizing this paradigm.

In contrast, other stress models typically use single stressors such as restraint, forced swimming (both physical stressors), or injection stress (a physical stressor if saline is injected or a physiological stress if an endotoxin such as LPS is injected). UCMS frequently incorporates these stressors (68% with restraint, 55% with forced swim), but studies using single stressors alone generally show less robust effects on cytokine levels. For example, restraint stress, used alone in 12% (*n* = 16) of studies, induced similar depression-like behaviours as UCMS however, non-significant changes between stressed and controls for IL-1β protein levels were reported 38% of the time, compared to UCMS in which non-significant results only were reported in 3% of studies. One potential explanation for the less robust effect of individual stressors could be a simple dose effect; if there are additive effects of multiple stressors, the more stressors used, the greater the impact on the individual. There is some evidence to support this hypothesis from studies using less prolonged restraint stress (lasting 2 h daily for 14 or 28 consecutive days) versus studies using extended daily durations or prolonged regimens over many more days. The less prolonged restraint models reported non-significant effects on depression-like behaviours whereas the more prolonged restraint models found significant effects on behaviour [54, 55]. Furthermore, studies employing more severe UCMS protocols, characterized by increased stress intensity or prolonged durations, elicited more robust cytokine alterations. For example, a UCMS regimen involving one stressor per day for 40 days [56] reported non-significant alterations in TNF-α protein levels. Conversely, a more intense protocol employing two stressors per day for 42 days [57] resulted in significantly elevated TNF-α protein levels.

An alternative explanation for the less robust effects of individual stressors is that different types of stressors can differentially alter both behavioural and biological outcomes, and that combining multiple stressors of the same type may not necessarily cause more severe outcomes [14]. For instance, restraint stress, primarily physical, might differ from psychological stressors in their effects. The CSDS model, while challenging to categorize as purely psychological, involves psychological stress and has shown greater variability in cytokine outcomes. Similar variability is seen in studies on maternal care deprivation. The absence of maternal care during early development has been shown to elicit psychological stress in offspring, however, the high rates of non-significant cytokine changes indicate a similar pattern to CSDS studies. Alternatively, it is conceivable that psychological stress may exert a greater influence on cytokine responses when combined with physical stressors, highlighting the potential interactive effects of different stress modalities. In combination, these findings indicate that further research is warranted to elucidate the nuanced interactions between psychological and physical stressors and their respective contributions to cytokine dysregulation in stress-related disorders. The main limitation in comparing stress types is that currently there are not enough studies in the literature that were designed to specifically test and compare stress types and research is warranted to elucidate the nuanced interactions between physical and psychological stressors and their respective contributions to cytokine dysregulation in stress-related disorders.

### Limitations of the literature

Limitations include the exclusion of depression models in which controls were vehicle injected. As previously discussed, this method could contribute to stress levels in control rodents, however, our sample size for comparison would have greatly increased, potentially enabling a more robust comparison between species types, sexes, and developmental stages. Further limitations stemmed from the poor reporting standards of fundamental characteristics of the rodent subjects, such as their genetic strain, age, weight, and sex which impacted on the reliability of comparisons. The absence of a standardized reporting framework for animal subjects was evident, leading to inconsistencies and conflicting results. Significantly, discrepancies were observed between reported body weight and age which did not align with standard strain characteristics as reported in growth charts from commercial suppliers (Supplementary Appendix 1), raising questions about the accuracy and reliability of the reported data. Moreover, among the studies examined in this review, four failed to disclose the sex of the rodents utilized. This limitation in reporting standards extended to key procedural details, including the determination of sample size, the implementation of randomization or blinding procedures, and the existence of criteria for the exclusion of animals, such as humane endpoints for those experiencing pain. Consequently, the poor reporting standards impact on the reliability of comparisons made between the studies in this review.

Additionally, our review identified a significant underreporting of animal housing conditions prior to and during experimentation (see Supplementary Data Collection Sheet) a factor crucial for understanding the welfare and behaviour of laboratory rodents. This is concerning as mice and rats are social animals, and social isolation is in itself a stressor [14, 58, 59]. Consequently, outcome measures derived from experiments involving singly housed rodents may be confounded by the stress induced by their housing conditions, in addition to the experimental stress procedures themselves, especially if the control groups were singly housed.

In addition to housing, the reporting of stress protocols within the reviewed studies was limited. Given the implications of stressor severity on biological and behavioural stress responses, it was surprising that stress procedures were frequently inadequately documented. For example, essential details such as the number of stress bouts, the location of the stress procedure, the rationale behind the choice of a particular stress type, and the occurrence of a pre-stress acclimation period were often omitted. Without essential information regarding the location, duration, and intensity of the stress procedure, we were only able to estimate the severity of the stress, making it difficult to accurately interpret and compare findings across studies. To minimize the inconsistency of reported measures, an effort should be made to utilize existing standards in animal research, such as the Animal Research: Reporting of In Vivo Experiments (ARRIVE) guidelines [60]. None of the studies included in this review managed to report the entirety of the ARRIVE Essential 10 (Fig. 2 and Supplemental ARRIVE guidelines checklist).

Furthermore, acknowledging the variability introduced by different cytokine measurement methods is crucial for interpreting results. Techniques such as qRT-PCR, RT-PCR, Western blot, and ELISA vary in sensitivity and in the type of molecules they assess, with some measuring RNA expression and others detecting protein levels. Notably, mRNA and protein levels do not always correlate due to post-transcriptional and post-translational regulation, as well as differences in assay sensitivity [61, 62]. Additionally, methodological differences in measuring the same target can impact result comparability across studies, contributing to inconsistencies in findings [63, 64]. Recognizing these limitations is essential for accurately interpreting stress-induced cytokine changes in MDD.

Another possible explanation for the variability in outcome measures observed, and further limitation, could be the inherent variability in stress response among individual animals. Rodents, like humans, may exhibit considerable individual differences in their physiological and psychological responses to stressors due to genetic predisposition, prior life experiences, and environmental influences. These inherent differences could confound the outcome measures and contribute to the variability observed.

### From rodents to humans: the importance of translation

Achieving a comprehensive understanding of the implications derived from animal research necessitates the ability to translate findings to human contexts. As shown in the Supplementary Data Collection Sheet, we aimed to classify stress types as either physical stress, psychological stress, physiological stress, or a combination of stressors however, categorizing various stressors into specific stress types was challenging. This problem was exacerbated due to the greater prevalence of UCMS studies and the relatively limited exploration of alternative stress paradigms within the field; as such, it was not possible to conduct rigorous comparison between stress types. Furthermore, inadequate reporting of the methodologies associated with stress induction exacerbated this challenge. Despite the limitations in reporting biases, this systematic review observed variations in stress-induced alterations, such as cytokine alterations across different developmental periods. Ideally, rodent studies should encompass measurements across various life stages, integrating stress inductions introduced at different time points, including early life stressors and those occurring later in life. In addition, there is a clear need to include both sexes in these studies. Such an approach enhances the translational relevance of findings to human populations, where stress exposure across the lifespan is diverse and multifaceted.

## Conclusion

This review highlights the need for a consensus on standardized methodologies to advance the understanding of how different stressors influence the inflammatory and behavioural profiles in rodent models of depression. Future research should address the identified gaps, particularly regarding sex differences and standardized methodologies, to build on these findings and enhance their translational potential. This would contribute to the broader goal of developing more precise and effective prevention and treatment strategies for major depressive disorder (MDD).

## Supplementary information

Supplemental text summary

Supplementary Appendix 1

Supplementary Appendix 2

Supplementary Table 1

Supplementary Table 2

Supplementary Table 3

Supplementary Table 4

Supplementary Table 5

Supplementary Table 6

Supplementary Table 7

Supplemental ARRIVE guidelines checklist.

Supplemental data extraction sheet.
