# Supplementary material for: Are behavioural and inflammatory profiles different according to type of stressor, developmental stage, and sex in rodent models of depression? A systematic review
Source: Mol Psychiatry. 2025 Aug 21;30(10):4971–82. doi: 10.1038/s41380-025-03138-2 (PMC12436165; doi:10.1038/s41380-025-03138-2)
Supplement: Supplementary file 1 — Supplemental text summary [file 41380_2025_3138_MOESM1_ESM.docx]

Supplementary information summary

1. Supplementary Appendix 1: Methods

**1.1 PRISMA checklist:** This file contains the PRISMA (Preferred Reporting Items for Systematic Reviews and Meta-Analyses guidelines) checklist, outlining the key reporting guidelines followed during the preparation of this systematic review.

**1.2 Search terms:** This file provides a detailed list of all search terms used in the systematic literature search, including Boolean operators and databases searched.

**1.3 Secondary outcome measures extracted from included publications:** This file contains the secondary outcome measures extracted from the included publications, detailing additional findings beyond the primary outcomes relevant to the systematic review.

**1.4 Abbreviations:** This file contains a list of abbreviations, and their corresponding definitions used throughout the systematic review to enhance clarity and readability.

**1.5 Growth charts:** This file contains growth charts used to estimate the age of rodents in studies where their age was not explicitly reported, based on weight.

1. Supplementary Appendix 2: Results

**Supplementary Table 1:** This file contains a summary of the characteristics of the studies included in this systematic review.

**Supplementary Table 2:** This file contains a comparison of outcome measures reported in studies using mice versus rats and male rodents versus female rodents.

**Supplementary Table 3:** This file contains a table showing the distribution of secondary outcome phenotypes linked to stress in adult rodent models.

**Supplementary Table 4:** This file contains a table showing the distribution of secondary outcome phenotypes linked to stress in adolescent rodent models.

**Supplementary Table 5:** This file contains a table showing the distribution of primary and secondary outcome phenotypes linked to stress in early postnatal rodent models.

**Supplementary Table 6:** This file contains a table showing the distribution of primary and secondary outcome phenotypes linked to maternal stress exposure in prenatal rodent models.

**Supplementary Table 7:** This file contains a comparison of outcome measures reported in studies that did not report sex of rodent subjects.
