# Supplementary material for: Are behavioural and inflammatory profiles different according to type of stressor, developmental stage, and sex in rodent models of depression? A systematic review
Source: Mol Psychiatry. 2025 Aug 21;30(10):4971–82. doi: 10.1038/s41380-025-03138-2 (PMC12436165; doi:10.1038/s41380-025-03138-2)
Supplement: Supplementary file 2 — Supplementary Appendix 1 [file 41380_2025_3138_MOESM2_ESM.docx]

**Supplementary Appendix 1**

**Methods**

- 1. Search terms

(“depression”[MeSH Terms] OR “depressive-like behaviour”[Abstract or Title] OR “depression-like behaviour”[Abstract or Title] AND “rat”[Abstract or Title] OR “mouse”[Abstract or Title] OR “mice” OR “rodent”[Abstract or Title] OR “mice”[Abstract or Title] AND “inflammatory”[MeSH Terms] OR “inflammation”[MeSH Terms] OR “inflammatory profile”[MeSH Terms] AND “stress”[All Fields] OR “stressor”[All Fields])

- 1. Secondary outcome measures extracted from included publications

Behavioural readouts attributed to depressive symptomology with the specific tests grouped according to key traits altered in depression: locomotor activity and anxiety (open field, elevated plus maze test, light/dark test), anhedonia (sucrose or saccharin preference test), behavioural despair (Porsolt forced swim test, tail suspension test) social dysfunction (social interaction test, social withdrawal test), cognitive impairment (Morris water maze, Y maze, novel object recognition, radial arm water maze), generalised helplessness (fear acquisition) and altered (poor) self-care (splash test, coat condition, grooming).

Measures of stress-related hormones (e.g., corticosterone, adrenocorticotropic hormone), brain metabolites (5-HT, dopamine, noradrenaline) and cellular phenotypes (neurogenesis and microglial activation).

- 1. Abbreviations

Stress types: CDS, chronic defeat stress; CMS, chronic mild stress; CSDS, chronic social defeat stress; CVS, chronic variable stress; RSDS, repeated social defeat stress; SDS, social defeat stress; UCMS, unpredictable chronic mild stress.

Behaviour: EPM, elevated plus maze test; FST, forced-swim test; OFT, open field test; SPT, sucrose preference test; TST, tail-suspension test.

Biological: 5-HIAA, 5-hydroxyindoleacetic acid; 5-HT, serotonin; ACTH, adrenocorticotropic hormone; CORT, corticosterone; CRH, corticotropin-releasing hormone; CXCL, CXC chemokine ligand; DA, dopamine; DOPAC, 3,4-dihydroxyphenylacetic acid; EPI, epinephrine; GFAP, glial fibrillary acidic protein; GSH, growth-stimulating hormone; H2S, hydrogen sulphide; HVA, homovanillic acid; Iba1, ionized calcium binding adaptor molecule 1; IL, interleukin; IFN, interferon; MCP, monocyte chemoattractant protein-1; MHPG, 3-methoxy-4-hydroxyphenylglycol; MDA, malondialdehyde; NE, norepinephrine; NO, nitric oxide; PGI2, prostaglandin I2; RNA, ribonucleic acid; TNF, tumour necrosis factor; TGF, transforming growth factor; TH, T helper; TxA2, thromboxane A2.

- 1. Growth charts

Growth Chart 1: BALB/c mice: A growth chart for BALB/cAnCrL inbred mice available from Charles River at https://www.criver.com/products-services/find-model/balbc-mouse?region=3671 CC BY-NC

Growth Chart 2: C57BL/6 mice: A growth chart for C57BL/6NCrL inbred mice available from Charles River at https://www.criver.com/products-services/find-model/c57bl6-mouse?region=3671 CC BY-NC

Growth Chart 3: CD-1 mice: A growth chart for CD-1 (ICR) outbred mice available from Charles River at https://www.criver.com/products-services/find-model/cd-1r-igs-mouse?region=3671 CC BY-NC

Growth Chart 4: Swiss Webster mice: A growth chart for Swiss Webster (CFW) outbred mice available from Charles River at https://www.criver.com/products-services/find-model/swiss-webster-cfw-mouse?region=3671 CC BY-NC

Growth Chart 5: Sprague Dawley rats: A growth chart for Sprague-Dawley outbred rats available from Charles River at https://www.criver.com/products-services/find-model/cd-sd-igs-rat?gclid=EAIaIQobChMI5ary-PeChgMV8bKDBx3xugorEAAYASAAEgIpFfD_BwE&region=3671 CC BY-NC

Growth Chart 6: Wistar rats: A growth chart for Wistar outbred rats available from Charles River at https://www.criver.com/products-services/find-model/wistar-igs-rat?region=3671 CC BY-NC
