# Supplementary material for: Are behavioural and inflammatory profiles different according to type of stressor, developmental stage, and sex in rodent models of depression? A systematic review
Source: Mol Psychiatry. 2025 Aug 21;30(10):4971–82. doi: 10.1038/s41380-025-03138-2 (PMC12436165; doi:10.1038/s41380-025-03138-2)
Supplement: Supplementary file 3 — Supplementary Appendix 2 [file 41380_2025_3138_MOESM3_ESM.docx]

**Supplementary Appendix 2**

**Results**

**2.1. Descriptive summary of secondary outcome phenotypes in adult rodents (Supplementary Table 3)**

Due to the range of reported secondary outcomes, we focused on measures that were reported in five or more studies (n ≥ 5). Assessment of depression-like behaviour in rodents frequently entails the utilization of standardized tests, including the sucrose preference test (SPT), known for gauging anhedonia-like symptoms, along with the forced swim test (FST) and/or the tail suspension test (TST), employed to assess indicators of despair-like behaviour. Notably, these depression-like manifestations were consistently identified, with a prevalence of 97%, as discerned from outcomes obtained through the SPT and the duration of immobility recorded in the TST. Furthermore, 93% (n = 41) of studies employing the FST reported increased immobility durations following stress induction. Moreover, heightened anxiety-like behaviour was evident in 78% (n = 32) of the reviewed studies that performed the OFT, and in 100% (n = 12) of the studies that used the EPM test. Additionally, declines in social behaviour and spatial learning/memory were documented in 100% (n = 11, n = 5, respectively) of studies. Any deviations from the above findings were due to non-significant changes in behaviour.

Various metabolic and neurotransmitter changes were documented following stress induction, the most frequently reported included serotonin (5-HT), corticosterone (CORT), dopamine (DA), and malondialdehyde (MDA). Specifically, increases were noted for CORT in 75% (n = 28) and MDA in 80% (n = 10) of the reviewed studies, respectively. Conversely, a significant decrease in 5-HT levels, as well as DA levels, were observed after stress exposure in adulthood, with reductions documented in 64% (n = 11) and 78% (n = 9) of reported cases, respectively.

Cellular measures constituted the third category of secondary outcomes scrutinized within this systematic review. Among the frequently reported outcomes were indicators of astrocyte activity, predominantly characterized by glial fibrillary acidic protein (GFAP), microglial activity, typically represented by allograft inflammatory factor 1 (Iba1), glucocorticoid receptor (GR) levels, neuronal proliferation, and oligodendrocyte markers, such as Olig2. Notably, within this category, microglial markers emerged as the most reported, with increased levels post stressor noted in 73% (n = 26) of the included studies. Conversely, a decline in measures of neuronal proliferation were demonstrated in 50% (n = 18) of the cases, while oligodendrocyte markers exhibited a decline in 83% (n = 18) of the studies. Astrocyte markers, though less frequently assessed, were reported in 12 studies, with half of them documenting a decrease. Additionally, GR levels exhibited a consistent reduction across all nine studies that reported this outcome measure.

**2.2. Descriptive summary of secondary outcome phenotypes in adolescent rodents (Supplementary Table 4)**

Consistent with investigations conducted on adult rodents, studies focusing on rodents exposed to stressors during adolescence similarly identified expressions of depression-like behaviour, as summarized in Supplementary Table 4.0. Depression-like behaviours were evaluated in all studies included in this review; most commonly by utilizing the FST and/or the TST and/or the SPT. Predominantly, reduced sucrose intake, indicative of anhedonia-like symptoms, was prevalent in 98% (n = 45) of studies utilizing the SPT. Similarly, signs of despair-like behaviour, characterized by increased immobility durations during the FST or TST, were reported in 95% (n = 43) or 93% (n = 28) of the studies, respectively. Alongside depression-like behaviours, anxiety-like manifestations based on findings from the OFT were documented across 32 studies, with 78% of studies demonstrating an increase in anxiety after stress induction. Additionally, cognitive decline and reduced social behaviour following stress exposure were consistently observed across all the studies assessing these behaviours.

Consistent with research involving adult rodent models, hormonal outcomes in adolescent rodents reported several notable findings, primarily concerning 5-HT, CORT, DA, and MDA. Among these, reductions in serotonin levels were reported in 83% (n = 12) of the cases investigated. Conversely, alterations in dopamine levels were less frequently observed, with reductions noted in only 33% (n = 6) of the cases reviewed. Additionally, CORT exhibited a significant increase in 85% (n = 13) of cases, while MDA levels were heightened across all seven studies.

Within this review, we noted a relative scarcity of reported cellular outcome measures in studies involving adolescent rodents compared to their adult counterparts. Nonetheless, akin to findings in adult rodent research, microglial markers emerged as a prominently investigated aspect. Among the 18 measures evaluated, 89% of cases reported heightened microglial marker levels following stress induction. Conversely, the reporting of astrocyte markers, predominantly assessed through glial fibrillary acidic protein (GFAP), presented a more varied pattern. Among the six studies examining astrocyte markers, only half (n = 3) reported decreased levels in response to stress, while the remainder documented increased levels. This discrepancy underscores the complexity of astrocytic involvement in stress-related neurobiology among adolescent rodents, necessitating further investigation to elucidate underlying mechanisms. Notably, most cellular measurements were derived from studies employing the UCMS paradigm, highlighting its prominence in elucidating neurobiological alterations associated with stress exposure in rodents. The singular exception to this trend was a solitary study employing a restraint stress model, which exclusively measured microglial marker levels.
