# Supplementary material for: Are behavioural and inflammatory profiles different according to type of stressor, developmental stage, and sex in rodent models of depression? A systematic review
Source: Mol Psychiatry. 2025 Aug 21;30(10):4971–82. doi: 10.1038/s41380-025-03138-2 (PMC12436165; doi:10.1038/s41380-025-03138-2)
Supplement: Supplementary file 4 — Supplementary Table 1 [file 41380_2025_3138_MOESM4_ESM.docx]

**Supplementary Table 1.**

Summary of the studies included in the systematic review (n = 130).

| Stress type | Species | Strain* | Sex | Life stage | (n) |
| --- | --- | --- | --- | --- | --- |
| UCMS/CVS/CMS | Mice | BALB/c | Males | Adulthood | 3 |
|  |  |  |  | Adolescence | 3 |
|  |  |  | Females | Adulthood | 1 |
|  |  | C57BL/6 | Males | Adulthood | 15 |
|  |  |  |  | Adolescence | 15 |
|  |  |  | Females | Adulthood | 2 |
|  |  |  |  | Adolescence | 1 |
|  |  |  | Both | Adulthood | 1 |
|  |  |  | Unknown | Adulthood | 1 |
|  |  |  |  | Adolescence | 1 |
|  |  | ICR | Males | Early postnatal | 5 |
|  |  | Krushinsky-Molodkina (KM) | Males | Adulthood | 2 |
|  |  | Swiss | Males | Adolescence | 1 |
|  | Rats | Sprague-Dawley | Males | Adulthood | 2 |
|  |  |  |  | Adolescence | 18 |
|  |  |  | Females | Adulthood | 1 |
|  |  | Wistar | Males | Adulthood | 4 |
|  |  |  |  | Adolescence | 8 |
|  |  |  | Females | Adulthood | 1 |
| Restraint stress | Mice | C57BL/6 | Males | Adulthood | 2 |
|  |  |  |  | Adolescence | 3 |
|  |  |  | Females | Adulthood | 2 |
|  |  |  | Unknown | Adolescence | 1 |
|  |  | ICR | Males | Early postnatal | 1 |
|  |  | Krushinsky-Molodkina (KM) | Males | Adulthood | 1 |
|  | Rats | Sprague-Dawley | Males | Adulthood | 1 |
|  |  |  |  | Adolescence | 2 |
|  |  |  | Females | Adulthood | 1 |
|  |  | Wistar | Males | Adulthood | 1 |
|  |  |  |  | Adolescence | 1 |
| CDS/CSDS/SDS/RSDS | Mice | C57BL/6 | Males | Adulthood | 8 |
|  |  |  |  | Adolescence | 3 |
|  | Rats | Sprague-Dawley | Males | Adolescence | 1 |
| Maternal stress * | Mice | C57BL/6 | Males | Early postnatal | 1 |
|  |  |  | Females | Early postnatal | 1 |
|  |  | CD-1 | Both | Adolescence | 1 |
|  | Rats | Sprague-Dawley | Males | Prenatal | 2 |
|  |  |  | Females | Adolescence | 1 |
|  |  | Wistar | Males | Adulthood | 1 |
|  |  |  | Both | Prenatal | 1 |
| Forced swim stress | Rats | Sprague-Dawley | Males | Adulthood | 1 |
|  |  |  | Females | Adulthood | 1 |
| Injection stress | Mice | C57BL/6 | Males | Adulthood | 1 |
|  |  | Swiss | Males | Adulthood | 1 |
|  | Rats | Sprague-Dawley | Males | Adulthood | 1 |
| Ultrasound stress | Mice | BALB/c | Males | Adulthood | 1 |
| Sleep deprivation stress | Mice | C57BL/6 | Males | Adulthood | 2 |
|  | Rats |  | Males | Early postnatal | 1 |

Studies using mice – 62% (n = 80); studies using rats – 38% (n = 50). Studies using male rodents (n = 112); studies using female rodents (n = 12); studies that combined findings from males and females (n = 3); studies that did not state the sex of the animals used (n = 3). * Studies using either maternal separation or maternal care deprivation were combined under the same heading of “Maternal Stress”.

Note: The number of studies exceeds the number of publications included in the review as several studies include multiple outcomes, such as investigations employing various versions of stress exposure but conducted within the same publication.

**Abbreviations**: CDS, chronic defeat stress; CMS, chronic mild stress; CSDS, chronic social defeat stress; CVS, chronic variable stress; KM, Krushinsky-Molodkina; SDS, social defeat stress; RSDS, repeated social defeat stress; UCMS, unpredictable chronic mild stress.

^*^Sub strain not listed as several articles did not include this information.
