# Supplementary material for: Are behavioural and inflammatory profiles different according to type of stressor, developmental stage, and sex in rodent models of depression? A systematic review
Source: Mol Psychiatry. 2025 Aug 21;30(10):4971–82. doi: 10.1038/s41380-025-03138-2 (PMC12436165; doi:10.1038/s41380-025-03138-2)
Supplement: Supplementary file 5 — Supplementary Table 2 [file 41380_2025_3138_MOESM5_ESM.docx]

**Supplementary Table 2.**

A comparison of studies utilizing mice (n = 80) versus rats (n = 50) and male rodents (n = 112) versus female rodents (n = 12).

| **Outcome measure** | **Result of outcome (n)**  ▴Significantly increased*  ▾Significantly decreased*  - No significant difference* | | | | | | | |
| --- | --- | --- | --- | --- | --- | --- | --- | --- |
|  |  |  |  |  |  |  |  |  |
|  |  |  |  |  |  |  |  |  |
|  | Mice | | Rats | | Males | | Females | |
|  | n | Result | n | Result | n | Result | n | Result |
| **Pro-inflammatory protein levels** |  |  |  |  |  |  |  |  |
| IL-1β | 69 (▴62) | ▴90% | 47 (▴43) | ▴91% | 98 (▴91) | ▴93% | 14 (▴10) | ▴71% |
| IL-6 | 55 (▴46) | ▴84% | 36 (▴33) | ▴92% | 86 (▴75) | ▴87% | 1 (▴1) | ▴100% |
| TNF-α | 69 (▴64) | ▴93% | 38 (▴35) | ▴92% | 99 (▴93) | ▴94% | 3 (▴2) | ▴67% |
| **Anti-inflammatory protein levels** |  |  |  |  |  |  |  |  |
| IL-10 | 13 (▾9) | ▾69% | 11 (▾7) | ▾64% | 20 (▾14) | ▾70% | 4 (▾2) | ▾50% |
| **Behavioural outcomes** |  |  |  |  |  |  |  |  |
| Anhedonia-like behaviour | 54 (▴52) | ▴96% | 36 (▴36) | ▴100% | 78 (▴76) | ▴97% | 8 (▴8) | ▴100% |
| Time immobile (FST) | 59 (▴55) | ▴93% | 35 (▴33) | ▴94% | 82 (▴78) | ▴95% | 6 (▴4) | ▴67% |
| Time immobile (TST) | 60 (▴58) | ▴97% | 5 (▴4) | ▴80% | 56 (▴55) | ▴98% | 5 (▴5) | ▴100% |
| Anxiety-like behaviour (OFT) | 43 (▴33) | ▴77% | 27 (▴21) | ▴78% | 57 (▴43) | ▴75% | 8 (▴7) | ▴88% |
| Anxiety-like behaviour (EPM) | 16 (▴14) | ▴88% | 13 (▴13) | ▴100% | 22 (▴21) | ▴95% | 6 (▴5) | ▴83% |
| Social behaviour | 12 (▾12) | ▾100% | 3 (▾1) | ▾33% | 13 (▾12) | ▾92% | 1 (▾0) | ▾0% |
| Spatial learning and memory | 4 (▾3) | ▾75% | 6 (▾6) | ▾100% | 7 (▾7) | ▾100% | 2 (▾1) | ▾50% |
| Recognition memory | 3 (▾3) | ▾100% | 1 (▾1) | ▾100% | 2 (▾2) | ▾100% | 1 (▾1) | ▾100% |
| **Hormones/**  **neurotransmitters** |  |  |  |  |  |  |  |  |
| 5-HT | 16 (▾12) | ▾75% | 12 (▾10) | ▾83% | 22 (▾18) | ▾82% | 4 (▾3) | ▾75% |
| CORT | 36 (▴28) | ▴78% | 13 (▴12) | ▴92% | 38 (▴31) | ▴82% | 7 (▴5) | ▴71% |
| DA | 13 (▾8) | ▾62% | 7 (▾6) | ▾86% | 15 (▾12) | ▾80% | 3 (▾2) | ▾67% |
| MDA | 11 (▴10) | ▴91% | 8 (▴7) | ▴88% | 19 (▴17) | ▴89% | 0 (▴0) | ▴0% |
| **Cellular outcomes** |  |  |  |  |  |  |  |  |
| Microglial markers | 36 (▴30) | ▴83% | 23 (▴19) | ▴83% | 50 (▴41) | ▴82% | 7 (▴6) | ▴86% |
| Astrocyte markers | 14 (▴6) | ▴43% | 7 (▴2) | ▴29% | 16 (▴8) | ▴50% | 3 (▴0) | ▴0% |

**Abbreviations**: Behaviour: EPM, elevated-plus maze test; FST, forced-swim test; OFT, open field test; TST, tail-suspension test. Biological: IL, interleukin; TNF, tumour necrosis factor; 5-HT, serotonin; CORT, corticosterone; DA, dopamine; MDA, malondialdehyde.

* Relative to stress-free control rodents
