# Supplementary material for: Are behavioural and inflammatory profiles different according to type of stressor, developmental stage, and sex in rodent models of depression? A systematic review
Source: Mol Psychiatry. 2025 Aug 21;30(10):4971–82. doi: 10.1038/s41380-025-03138-2 (PMC12436165; doi:10.1038/s41380-025-03138-2)
Supplement: Supplementary file 6 — Supplementary Table 3 [file 41380_2025_3138_MOESM6_ESM.docx]

**Supplementary Table 3.**

The secondary outcome phenotypes associated with stress exposure in adult rodents (n = 59).

| **Outcome measure** | **Total number of studies measuring the outcome of interest (% total studies with specific outcome of significant increase** ▴ **or decrease** ▾**)** | **Number of studies with specific outcome of**  ▴Significantly increased*  ▾Significantly decreased*  - No significant difference* | | | | | | | | | | | | | | |  |
| --- | --- | --- | --- | --- | --- | --- | --- | --- | --- | --- | --- | --- | --- | --- | --- | --- | --- |
|  |  | UCMS/CMS/ CVS (n = 33) | | | Restraint stress  (n = 8) | | | CSDS/SDS/  RSDS (n = 9) | | | Maternal stress (n = 1) | | | Other types of  stress (n = 8) | | | |
|  |  | ▴ | ▾ | - | ▴ | ▾ | - | ▴ | ▾ | - | ▴ | ▾ | - | ▴ | ▾ | - | |
| **Behavioural outcomes** |  |  |  |  |  |  |  |  |  |  |  |  |  |  |  |  | |
| Anhedonia-like behaviour | 38 (97%▴) | 22 | 0 | 0 | 6 | 0 | 0 | 4 | 0 | 0 | 0 | 0 | 0 | 5 | 0 | 1 | |
| Time immobile (FST) | 41 (93%▴) | 22 | 0 | 0 | 4 | 0 | 3 | 5 | 0 | 0 | 1 | 0 | 0 | 6 | 0 | 0 | |
| Time immobile (TST) | 30 (97%▴) | 21 | 0 | 0 | 3 | 0 | 0 | 2 | 0 | 0 | 0 | 0 | 0 | 3 | 0 | 1 | |
| Defensive behaviour | 1 (100%▴) | 0 | 0 | 0 | 0 | 0 | 0 | 0 | 0 | 0 | 0 | 0 | 0 | 1 | 0 | 0 | |
| Anxiety-like behaviour  (OFT) | 32 (78%▴) | 16 | 0 | 4 | 5 | 0 | 1 | 3 | 0 | 0 | 0 | 0 | 1 | 1 | 0 | 1 | |
| Anxiety-like behaviour (EPM) | 12 (100%▴) | 9 | 0 | 0 | 1 | 0 | 0 | 1 | 0 | 0 | 0 | 0 | 0 | 1 | 0 | 0 | |
| Spatial learning/memory | 5 (100%▾) | 0 | 4 | 0 | 0 | 0 | 0 | 0 | 1 | 0 | 0 | 0 | 0 | 0 | 0 | 0 | |
| Recognition memory | 2 (100%▾) | 0 | 2 | 0 | 0 | 0 | 0 | 0 | 0 | 0 | 0 | 0 | 0 | 0 | 0 | 0 | |
| Social behaviour | 11 (100%▾) | 0 | 2 | 0 | 0 | 1 | 0 | 0 | 8 | 0 | 0 | 0 | 0 | 0 | 0 | 0 | |
| Grooming status | 4 (100%▾) | 0 | 4 | 0 | 0 | 0 | 0 | 0 | 0 | 0 | 0 | 0 | 0 | 0 | 0 | 0 | |
| Fear acquisition | 1 (100%▴) | 0 | 0 | 0 | 0 | 0 | 0 | 1 | 0 | 0 | 0 | 0 | 0 | 0 | 0 | 0 | |
| Motor activity | 1 (100%▾) | 0 | 0 | 0 | 0 | 0 | 0 | 0 | 1 | 0 | 0 | 0 | 0 | 0 | 0 | 0 | |
| **Hormones/metabolites**  **neurotransmitters** |  |  |  |  |  |  |  |  |  |  |  |  |  |  |  |  | |
| 3-nitrotyrosine | 1 (100%▴) | 0 | 0 | 0 | 0 | 0 | 0 | 0 | 0 | 0 | 0 | 0 | 0 | 1 | 0 | 0 | |
| 5-HIAA | 6 (17%▾) | 0 | 0 | 3 | 0 | 1 | 1 | 0 | 0 | 0 | 0 | 0 | 0 | 1 | 0 | 0 | |
| 5-HT | 11 (64%▾) | 0 | 5 | 2 | 0 | 0 | 2 | 0 | 0 | 0 | 0 | 0 | 0 | 0 | 2 | 0 | |
| 8-isoprostane | 1 (100%▴) | 0 | 0 | 0 | 0 | 0 | 0 | 1 | 0 | 0 | 0 | 0 | 0 | 0 | 0 | 0 | |
| ACTH | 7 (71%▴) | 4 | 0 | 0 | 1 | 0 | 2 | 0 | 0 | 0 | 0 | 0 | 0 | 0 | 0 | 0 | |
| CORT | 28 (75%▴) | 14 | 0 | 0 | 3 | 0 | 3 | 1 | 0 | 3 | 0 | 0 | 0 | 3 | 0 | 1 | |
| CRH | 5 (20%▴) | 1 | 0 | 1 | 0 | 0 | 3 | 0 | 0 | 0 | 0 | 0 | 0 | 0 | 0 | 0 | |
| DA | 9 (78%▾) | 0 | 5 | 1 | 0 | 1 | 1 | 0 | 0 | 0 | 0 | 0 | 0 | 0 | 1 | 0 | |
| DOPAC | 5 (40%▾) | 0 | 1 | 2 | 0 | 1 | 1 | 0 | 0 | 0 | 0 | 0 | 0 | 0 | 0 | 0 | |
| GABA | 1 (100%▾) | 0 | 0 | 0 | 0 | 1 | 0 | 0 | 0 | 0 | 0 | 0 | 0 | 0 | 0 | 0 | |
| GSH | 4 (75%▴) | 3 | 0 | 0 | 0 | 0 | 0 | 0 | 0 | 0 | 0 | 0 | 0 | 0 | 1 | 0 | |
| H2S | 1 (100%▾) | 0 | 1 | 0 | 0 | 0 | 0 | 0 | 0 | 0 | 0 | 0 | 0 | 0 | 0 | 0 | |
| HVA | 4 (25%▾) | 0 | 1 | 1 | 0 | 0 | 2 | 0 | 0 | 0 | 0 | 0 | 0 | 0 | 0 | 0 | |
| MHPG | 3 (0%▾) | 0 | 0 | 3 | 0 | 0 | 0 | 0 | 0 | 0 | 0 | 0 | 0 | 0 | 0 | 0 | |
| MDA | 10 (80%▴) | 5 | 0 | 0 | 1 | 1 | 0 | 0 | 0 | 0 | 0 | 0 | 0 | 2 | 0 | 1 | |
| NE | 8 (0%▴) | 0 | 3 | 2 | 0 | 1 | 1 | 0 | 0 | 0 | 0 | 0 | 0 | 0 | 1 | 0 | |
| Nitrite | 1 | 0 | 0 | 1 | 0 | 0 | 0 | 0 | 0 | 0 | 0 | 0 | 0 | 0 | 0 | 0 | |
| NO | 2 (0%▴) | 0 | 2 | 0 | 0 | 0 | 0 | 0 | 0 | 0 | 0 | 0 | 0 | 0 | 0 | 0 | |
| PGE2 | 1 (100%▴) | 1 | 0 | 0 | 0 | 0 | 0 | 0 | 0 | 0 | 0 | 0 | 0 | 0 | 0 | 0 | |
| PGI2 | 2 (100%▾) | 0 | 2 | 0 | 0 | 0 | 0 | 0 | 0 | 0 | 0 | 0 | 0 | 0 | 0 | 0 | |
| TxA2 | 2 (100%▴) | 2 | 0 | 0 | 0 | 0 | 0 | 0 | 0 | 0 | 0 | 0 | 0 | 0 | 0 | 0 | |
| **Cellular outcome** |  |  |  |  |  |  |  |  |  |  |  |  |  |  |  |  | |
| Apoptosis rate | 1 (100%▴) | 1 | 0 | 0 | 0 | 0 | 0 | 0 | 0 | 0 | 0 | 0 | 0 | 0 | 0 | 0 | |
| Apoptotic markers | 2 (100%▴) | 0 | 0 | 0 | 0 | 0 | 0 | 0 | 0 | 0 | 0 | 0 | 0 | 2 | 0 | 0 | |
| Astrocyte marker (GFAP) | 11 (45%▾) | 1 | 5 | 3 | 0 | 0 | 0 | 0 | 0 | 2 | 0 | 0 | 0 | 0 | 0 | 0 | |
| GR levels | 9 (100%▾) | 0 | 6 | 0 | 0 | 3 | 0 | 0 | 0 | 0 | 0 | 0 | 0 | 0 | 0 | 0 | |
| Hippocampal cell count | 3 (67%▾) | 0 | 2 | 1 | 0 | 0 | 0 | 0 | 0 | 0 | 0 | 0 | 0 | 0 | 0 | 0 | |
| Hippocampal spine density | 1 (100%▾) | 0 | 0 | 0 | 0 | 0 | 0 | 0 | 1 | 0 | 0 | 0 | 0 | 0 | 0 | 0 | |
| Microglial marker (Iba1) | 26 (73%▴) | 13 | 0 | 5 | 3 | 0 | 0 | 1 | 0 | 2 | 0 | 0 | 0 | 2 | 0 | 0 | |
| Microglial cell count | 2 (100%▴) | 2 | 0 | 0 | 0 | 0 | 0 | 0 | 0 | 0 | 0 | 0 | 0 | 0 | 0 | 0 | |
| Neuronal proliferation | 18 (50%▾) | 1 | 8 | 3 | 4 | 1 | 1 | 0 | 0 | 0 | 0 | 0 | 0 | 0 | 0 | 0 | |
| Oligodendrocyte markers | 18 (83%▾) | 0 | 12 | 0 | 2 | 3 | 1 | 0 | 0 | 0 | 0 | 0 | 0 | 0 | 0 | 0 | |

Note: The number of studies exceeds the number of publications included in the review as several studies include multiple outcomes, such as investigations employing various versions of stress exposure but conducted within the same publication.

**Abbreviations**: Stress types: CDS, chronic defeat stress; CMS, chronic mild stress; CSDS, chronic social defeat stress; CVS, chronic variable stress; RSDS, repeated social defeat stress; SDS, social defeat stress; UCMS, unpredictable chronic mild stress. Behavioural: EPM, elevated plus maze test; FST, forced-swim test; OFT, open field test; TST, tail-suspension test. Biological: 5-HIAA, 5-hydroxyindoleacetic acid; 5-HT, serotonin; ACTH, adrenocorticotropic hormone; CORT, corticosterone; CRH, corticotropin-releasing hormone; DA, dopamine; DOPAC, 3,4-Dihydroxyphenylacetic acid; GABA, gamma-aminobutyric acid; GR, Glucocorticoid receptors; GSH, growth-stimulating hormone; H2S, hydrogen sulphide; HVA, homovanillic acid; MHPG, 3-Methoxy-4-hydroxyphenylglycol; MDA, malondialdehyde; NE, norepinephrine; NO, nitric oxide; PGE2, prostaglandin E2; PGI2, prostaglandin I2; TxA2, thromboxane A2.

Maternal stress includes maternal care deprivation (n = 1).

Other stress types include injection stress (n = 3), sleep deprivation stress (n = 2), forced swim stress (n = 2), ultrasound stress (n = 1).

* Relative to stress-free control rodents
