# Supplementary material for: Are behavioural and inflammatory profiles different according to type of stressor, developmental stage, and sex in rodent models of depression? A systematic review
Source: Mol Psychiatry. 2025 Aug 21;30(10):4971–82. doi: 10.1038/s41380-025-03138-2 (PMC12436165; doi:10.1038/s41380-025-03138-2)
Supplement: Supplementary file 7 — Supplementary Table 4 [file 41380_2025_3138_MOESM7_ESM.docx]

**Supplementary Table 4**.

The secondary outcome phenotypes associated with stress exposure in adolescent rodents (n = 59).

| **Outcome measure** | **Total number of studies measuring the outcome of interest (% total studies with specific outcome of significant increase** ▴ **or decrease** ▾**)** | **Number of studies with specific outcome of**  ▴Significantly increased*  ▾Significantly decreased*  - No significant difference* | | | | | | | | | | | |
| --- | --- | --- | --- | --- | --- | --- | --- | --- | --- | --- | --- | --- | --- |
|  |  | UCMS/CMS/ CVS  (n = 47) | | | Restraint Stress  (n = 7) | | | CSDS/SDS/RSDS  (n = 3) | | | Maternal  Stress (n = 2) | | |
|  |  | ▴ | ▾ | - | ▴ | ▾ | - | ▴ | ▾ | - | ▴ | ▾ | - |
| **Behavioural outcomes** |  |  |  |  |  |  |  |  |  |  |  |  |  |
| Anhedonia-like behaviour | 45 (▴98%) | 38 | 0 | 0 | 4 | 0 | 0 | 1 | 0 | 1 | 1 | 0 | 0 |
| Time immobile (FST) | 43 (▴95%) | 34 | 0 | 2 | 4 | 0 | 0 | 1 | 0 | 0 | 2 | 0 | 0 |
| Time immobile (TST) | 28 (▴93%) | 23 | 0 | 1 | 2 | 0 | 1 | 0 | 0 | 0 | 1 | 0 | 0 |
| Anxiety-like behaviour (OFT) | 32 (▴78%) | 21 | 0 | 6 | 3 | 0 | 0 | 0 | 0 | 0 | 1 | 0 | 1 |
| Anxiety-like behaviour (EPM) | 14 (▴93%) | 11 | 0 | 1 | 1 | 0 | 0 | 0 | 0 | 0 | 1 | 0 | 0 |
| Alcohol consumption | 1 (▴100%) | 0 | 0 | 0 | 0 | 0 | 0 | 1 | 0 | 0 | 0 | 0 | 0 |
| Spatial learning and memory | 4 (▾100%) | 0 | 2 | 0 | 0 | 0 | 0 | 0 | 0 | 0 | 0 | 2 | 0 |
| Recognition memory | 1 (▾100%) | 0 | 0 | 0 | 0 | 0 | 0 | 0 | 0 | 0 | 0 | 1 | 0 |
| Social behaviour | 3 (▾100%) | 0 | 1 | 0 | 0 | 0 | 0 | 0 | 2 | 0 | 0 | 0 | 0 |
| Grooming status | 1 (▾0%) | 0 | 0 | 1 | 0 | 0 | 0 | 0 | 0 | 0 | 0 | 0 | 0 |
| Passive avoidance | 1 (▴100%) | 1 | 0 | 0 | 0 | 0 | 0 | 0 | 0 | 0 | 0 | 0 | 0 |
| **Hormones/**  **metabolites** |  |  |  |  |  |  |  |  |  |  |  |  |  |
| 5-HIAA | 3 (▾67%) | 0 | 1 | 0 | 0 | 1 | 1 | 0 | 0 | 0 | 0 | 0 | 0 |
| 5-HT | 14 (▾86%) | 0 | 11 | 1 | 0 | 1 | 1 | 0 | 0 | 0 | 0 | 0 | 0 |
| ACTH | 3 (▴67%) | 2 | 1 | 0 | 0 | 0 | 0 | 0 | 0 | 0 | 0 | 0 | 0 |
| CORT | 17 (▴88%) | 13 | 0 | 1 | 1 | 0 | 1 | 1 | 0 | 0 | 0 | 0 | 0 |
| CRH | 3 (▴67%) | 2 | 0 | 1 | 0 | 0 | 0 | 0 | 0 | 0 | 0 | 0 | 0 |
| DA | 9 (▾44%) | 0 | 4 | 2 | 1 | 0 | 2 | 0 | 0 | 0 | 0 | 0 | 0 |
| DOPAC | 2 (▾0%) | 0 | 0 | 0 | 0 | 0 | 2 | 0 | 0 | 0 | 0 | 0 | 0 |
| EPI | 2 (▴50%) | 0 | 0 | 0 | 0 | 0 | 1 | 1 | 0 | 0 | 0 | 0 | 0 |
| GSH | 3 (▾100%) | 0 | 3 | 0 | 0 | 0 | 0 | 0 | 0 | 0 | 0 | 0 | 0 |
| L-DOPA | 1 (▾100%) | 0 | 1 | 0 | 0 | 0 | 0 | 0 | 0 | 0 | 0 | 0 | 0 |
| MDA | 10 (▴100%) | 9 | 0 | 0 | 1 | 0 | 0 | 0 | 0 | 0 | 0 | 0 | 0 |
| NE | 3 (▾100%) | 0 | 3 | 0 | 0 | 0 | 0 | 0 | 0 | 0 | 0 | 0 | 0 |
| NO | 2 (▴100%) | 2 | 0 | 0 | 0 | 0 | 0 | 0 | 0 | 0 | 0 | 0 | 0 |
| **Cellular outcome** |  |  |  |  |  |  |  |  |  |  |  |  |  |
| Apoptosis rate | 2 (▴100%) | 2 | 0 | 0 | 0 | 0 | 0 | 0 | 0 | 0 | 0 | 0 | 0 |
| Astrocyte marker | 10 (▾43%) | 7 | 3 | 0 | 0 | 0 | 0 | 0 | 0 | 0 | 0 | 0 | 0 |
| Hippocampal cell count | 2 (▾100%) | 0 | 2 | 0 | 0 | 0 | 0 | 0 | 0 | 0 | 0 | 0 | 0 |
| Hippocampal spine density | 1 (▾100%) | 0 | 1 | 0 | 0 | 0 | 0 | 0 | 0 | 0 | 0 | 0 | 0 |
| Microglial markers | 26 (▴96%) | 23 | 0 | 1 | 1 | 0 | 0 | 0 | 0 | 0 | 1 | 0 | 0 |
| Neuronal proliferation | 2 (▾100%) | 0 | 2 | 0 | 0 | 0 | 0 | 0 | 0 | 0 | 0 | 0 | 0 |
| Synapse count | 1 (▾100%) | 0 | 1 | 0 | 0 | 0 | 0 | 0 | 0 | 0 | 0 | 0 | 0 |

Note: The number of studies exceeds the number of publications included in the review as several studies include multiple outcomes, such as investigations employing various versions of stress exposure but conducted within the same publication.

**Abbreviations:** Stress types: CDS, chronic defeat stress; CMS, chronic mild stress; CSDS, chronic social defeat stress; CVS, chronic variable stress; RSDS, repeated social defeat stress; SDS, social defeat stress; UCMS, unpredictable chronic mild stress. Behavioural: EPM, elevated plus maze test; FST, forced-swim test; OFT, open field test; TST, tail-suspension test. Biological: 5-HIAA, 5-hydroxyindoleacetic acid; 5-HT, serotonin; ACTH, adrenocorticotropic hormone; CORT, corticosterone; CRH, corticotropin-releasing hormone; DA, dopamine; DOPAC, 3,4-Dihydroxyphenylacetic acid; EPI, epinephrine; GSH, growth-stimulating hormone; L-DOPA, levodopa or l-3,4-dihydroxyphenylalanine; MDA, malondialdehyde; NE, norepinephrine; NO, nitric oxide.

Maternal stress includes maternal care deprivation (n = 2).

* Relative to stress-free control rodents
