# Supplementary material for: Are behavioural and inflammatory profiles different according to type of stressor, developmental stage, and sex in rodent models of depression? A systematic review
Source: Mol Psychiatry. 2025 Aug 21;30(10):4971–82. doi: 10.1038/s41380-025-03138-2 (PMC12436165; doi:10.1038/s41380-025-03138-2)
Supplement: Supplementary file 8 — Supplementary Table 5 [file 41380_2025_3138_MOESM8_ESM.docx]

**Supplementary Table 5**.

Primary and secondary outcome phenotypes associated with stress exposure in early postnatal rodents (n = 9).

| **Outcome measure** | **Total number of studies measuring the outcome of interest (% total studies with specific outcome of significant increase** ▴ **or decrease** ▾**)** | **Result of the outcome (n)**  ▴Significantly increased*  ▾Significantly decreased*  - No significant difference* | | | | | | | | | | | | |  |  |
| --- | --- | --- | --- | --- | --- | --- | --- | --- | --- | --- | --- | --- | --- | --- | --- | --- |
|  |  | UCMS/CMS/CVS  (n = 5) | | | Restraint stress  (n = 1) | | | Maternal care deprivation (n = 2) | | | | Sleep deprivation  (n = 1) | | | | |
|  |  | ▴ | ▾ | - | ▴ | ▾ | - | ▴ | ▾ | - | ▴ | | ▾ | - | |  |
| **Pro-inflammatory cytokines – protein levels** |  |  |  |  |  |  |  |  |  |  |  | |  |  | |  |
| IL-1β | 7 (86%▴) | 6 | 0 | 0 | 0 | 0 | 0 | 0 | 0 | 1 | 0 | | 0 | 0 | |  |
| IL-6 | 6 (100%▴) | 5 | 0 | 0 | 0 | 0 | 0 | 0 | 0 | 0 | 1 | | 0 | 0 | |  |
| CXCL1 | 1 (0%▴) | 0 | 0 | 0 | 0 | 0 | 0 | 0 | 0 | 1 | 0 | | 0 | 0 | |  |
| TNF-α | 9 (89%▴) | 6 | 0 | 0 | 1 | 0 | 0 | 0 | 0 | 1 | 1 | | 0 | 0 | |  |
| **Pro-inflammatory cytokines – RNA levels** |  |  |  |  |  |  |  |  |  |  |  | |  |  | |  |
| IL-1β | 1 (100%▴) | 1 | 0 | 0 | 0 | 0 | 0 | 0 | 0 | 0 | 0 | | 0 | 0 | |  |
| IL-6 | 1 (100%▴) | 1 | 0 | 0 | 0 | 0 | 0 | 0 | 0 | 0 | 0 | | 0 | 0 | |  |
| TNF-α | 1 (100%▴) | 1 | 0 | 0 | 0 | 0 | 0 | 0 | 0 | 0 | 0 | | 0 | 0 | |  |
| **Anti-inflammatory cytokines – protein levels** |  |  |  |  |  |  |  |  |  |  |  | |  |  | |  |
| IL-10 | 5 (60%▾) | 0 | 0 | 0 | 0 | 0 | 0 | 0 | 3 | 2 | 0 | | 0 | 0 | |  |
| TGF-β | 1 (100%▾) | 0 | 0 | 0 | 0 | 1 | 0 | 0 | 0 | 0 | 0 | | 0 | 0 | |  |
| **TH2 cytokines – protein levels** |  |  |  |  |  |  |  |  |  |  |  | |  |  | |  |
| IL-5 | 1 (0%▾) | 0 | 0 | 0 | 0 | 0 | 0 | 0 | 0 | 1 | 0 | | 0 | 0 | |  |
| **Behavioural outcomes** |  |  |  |  |  |  |  |  |  |  |  | |  |  | |  |
| Anhedonia-like behaviour | 5 (100%▴) | 5 | 0 | 0 | 0 | 0 | 0 | 0 | 0 | 0 | 0 | | 0 | 0 | |  |
| Time immobile (FST) | 8 (88%▴) | 5 | 0 | 0 | 1 | 0 | 0 | 1 | 0 | 0 | 0 | | 0 | 1 | |  |
| Time immobile (TST) | 7 (100%▴) | 5 | 0 | 0 | 1 | 0 | 0 | 1 | 0 | 0 | 0 | | 0 | 0 | |  |
| Anxiety-like behaviour (OFT) | 8 (38%▴) | 2 | 0 | 1 | 1 | 0 | 1 | 0 | 0 | 2 | 0 | | 0 | 1 | |  |
| Anxiety-like behaviour (EPM) | 3 (67%▴) | 0 | 0 | 0 | 1 | 0 | 0 | 0 | 0 | 1 | 1 | | 0 | 0 | |  |
| Spatial learning and memory | 1 (0%▾) | 0 | 0 | 0 | 0 | 0 | 0 | 0 | 0 | 1 | 0 | | 0 | 0 | |  |
| Recognition memory | 1 (100%▾) | 0 | 0 | 0 | 0 | 0 | 0 | 0 | 1 | 0 | 0 | | 0 | 0 | |  |
| Social behaviour | 1 (0%▾) | 0 | 0 | 0 | 0 | 0 | 0 | 0 | 0 | 0 | 0 | | 0 | 1 | |  |
| **Hormones/**  **metabolites** |  |  |  |  |  |  |  |  |  |  |  | |  |  | |  |
| 5-HT | 3 (100%▾) | 0 | 3 | 0 | 0 | 0 | 0 | 0 | 0 | 0 | 0 | | 0 | 0 | |  |
| ACTH | 1 (100%▴) | 1 | 0 | 0 | 0 | 0 | 0 | 0 | 0 | 0 | 0 | | 0 | 0 | |  |
| CORT | 3 (100%▴) | 3 | 0 | 0 | 0 | 0 | 0 | 0 | 0 | 0 | 0 | | 0 | 0 | |  |
| CRF | 1 (100%▴) | 1 | 0 | 0 | 0 | 0 | 0 | 0 | 0 | 0 | 0 | | 0 | 0 | |  |
| DA | 2 (100%▾) | 0 | 2 | 0 | 0 | 0 | 0 | 0 | 0 | 0 | 0 | | 0 | 0 | |  |
| GSH | 1 (100%▾) | 0 | 1 | 0 | 0 | 0 | 0 | 0 | 0 | 0 | 0 | | 0 | 0 | |  |
| MDA | 1 (100%▴) | 1 | 0 | 0 | 0 | 0 | 0 | 0 | 0 | 0 | 0 | | 0 | 0 | |  |
| NE | 2 (100%▾) | 0 | 2 | 0 | 0 | 0 | 0 | 0 | 0 | 0 | 0 | | 0 | 0 | |  |
| **Cellular outcomes** |  |  |  |  |  |  |  |  |  |  |  | |  |  | |  |
| Microglial markers | 5 (60%▴) | 0 | 0 | 0 | 0 | 0 | 0 | 3 | 0 | 2 | 0 | | 0 | 0 | |  |

Studies using mice – 89% (n = 8); studies using rats – 11% (n = 1).

Note: The number of studies exceeds the number of publications included in the review as several studies include multiple outcomes, such as investigations employing various versions of stress exposure but conducted within the same publication.

**Abbreviations:** Stressors: UCMS, unpredictable chronic mild stress; CVS, chronic variable stress; CMS, chronic mild stress; CDS, chronic defeat stress; CSDS, chronic social defeat stress; SDS, social defeat stress; RSDS, repeated social defeat stress. Behaviour: EPM, elevated plus maze test; FST, forced-swim test; OFT, open field test; TST, tail-suspension test. Biological*:* 5-HT, serotonin; ACTH, adrenocorticotropic hormone; CORT, corticosterone; CRF, corticotropin releasing factor; CXCL, CXC chemokine ligand; DA, dopamine; GSH, growth-stimulating hormone; IL, interleukin; MDA, malondialdehyde; NE, norepinephrine; RNA, ribonucleic acid; TGF, transforming growth factor; TH, T helper; TNF, tumour necrosis factor.
