# Supplementary material for: Are behavioural and inflammatory profiles different according to type of stressor, developmental stage, and sex in rodent models of depression? A systematic review
Source: Mol Psychiatry. 2025 Aug 21;30(10):4971–82. doi: 10.1038/s41380-025-03138-2 (PMC12436165; doi:10.1038/s41380-025-03138-2)
Supplement: Supplementary file 9 — Supplementary Table 6 [file 41380_2025_3138_MOESM9_ESM.docx]

**Supplementary Table 6.**

Breakdown of primary and secondary outcome phenotypes associated with maternal stress exposure in prenatal rodents (n = 3).

| **Outcome measure** | **Total number of studies measuring the outcome of interest (% total studies with specific outcome of significant increase** ▴ **or decrease** ▾**)** | **Result of outcome (n)**  ▴Significantly increased*  ▾Significantly decreased*  - No significant difference* | | | | | | | | |  |
| --- | --- | --- | --- | --- | --- | --- | --- | --- | --- | --- | --- |
|  |  | Maternal UCMS  (n = 1) | | | Maternal light exposure (n = 1) | | | Maternal restraint  (n = 1) | | | |
|  |  | ▴ | ▾ | - | ▴ | ▾ | - | ▴ | ▾ | - | |
| **Pro-inflammatory cytokines – protein levels** |  |  |  |  |  |  |  |  |  |  | |
| CCL2 | 1 (100%▴) | 0 | 0 | 0 | 1 | 0 | 0 | 0 | 0 | 0 | |
| CXCL12 | 1 (100%▴) | 0 | 0 | 0 | 1 | 0 | 0 | 0 | 0 | 0 | |
| IL-1β | 2 (100%▴) | 1 | 0 | 0 | 1 | 0 | 0 | 0 | 0 | 0 | |
| IL-6 | 1 (100%▴) | 0 | 0 | 0 | 1 | 0 | 0 | 0 | 0 | 0 | |
| IL-18 | 2 (100%▴) | 1 | 0 | 0 | 1 | 0 | 0 | 0 | 0 | 0 | |
| TNF-α | 1 (100%▴) | 0 | 0 | 0 | 1 | 0 | 0 | 0 | 0 | 0 | |
| **Pro-inflammatory cytokines – RNA levels** |  |  |  |  |  |  |  |  |  |  | |
| CCL2 | 1 (100%▴) | 0 | 0 | 0 | 1 | 0 | 0 | 0 | 0 | 0 | |
| CXCL12 | 1 (0%▴) | 0 | 0 | 0 | 0 | 0 | 1 | 0 | 0 | 0 | |
| IL-1β | 5 (20%▴) | 0 | 0 | 0 | 1 | 0 | 0 | 0 | 0 | 4 | |
| IL-6 | 5 (20%▴) | 0 | 0 | 0 | 1 | 0 | 0 | 0 | 0 | 4 | |
| IL-18 | 1 (100%▴) | 0 | 0 | 0 | 1 | 0 | 0 | 0 | 0 | 0 | |
| TNF-α | 4 (20%▴) | 0 | 0 | 0 | 1 | 0 | 0 | 0 | 1 | 3 | |
| **Behavioural outcomes** |  |  |  |  |  |  |  |  |  |  | |
| Anhedonia-like behaviour | 2 (100%▴) | 1 | 0 | 0 | 1 | 0 | 0 | 0 | 0 | 0 | |
| Time immobile (FST) | 2 (100%▴) | 1 | 0 | 0 | 1 | 0 | 0 | 0 | 0 | 0 | |
| Anxiety-like behaviour (OFT) | 2 (100%▴) | 1 | 0 | 0 | 0 | 0 | 0 | 0 | 0 | 0 | |
| Anxiety-like behaviour (EPM) | 1 (100%▴) | 0 | 0 | 0 | 0 | 0 | 0 | 1 | 0 | 0 | |
| **Hormones/**  **metabolites** |  |  |  |  |  |  |  |  |  |  | |
| CORT | 1 (100%▴) | 1 | 0 | 0 | 0 | 0 | 0 | 0 | 0 | 0 | |
| NO | 1 (100%▴) | 0 | 0 | 0 | 1 | 0 | 0 | 0 | 0 | 0 | |

Studies using rats – 100% (n = 3).

Note: The number of studies exceeds the number of publications included in the review as several studies include multiple outcomes, such as investigations employing various versions of stress exposure but conducted within the same publication.

**Abbreviations:** Biological: CCL, chemokine ligand; CORT, corticosterone; CXCL, CXC chemokine ligand; IL, interleukin; NO, nitric oxide; RNA, ribonucleic acid; TNF, tumour necrosis factor. Behavioural: EPM, elevated plus maze test; FST, forced-swim test; OFT, open field test.

* Relative to stress-free control rodents
