# Supplementary material for: Are behavioural and inflammatory profiles different according to type of stressor, developmental stage, and sex in rodent models of depression? A systematic review
Source: Mol Psychiatry. 2025 Aug 21;30(10):4971–82. doi: 10.1038/s41380-025-03138-2 (PMC12436165; doi:10.1038/s41380-025-03138-2)
Supplement: Supplementary file 10 — Supplementary Table 7 [file 41380_2025_3138_MOESM10_ESM.docx]

**Supplementary Table 7.**

A comparison of studies that did not report sex of rodent subjects (n = 3).

| **Outcome measure** | **Total number of studies measuring the outcome of interest (% total studies with specific outcome of significant increase** ▴ **or decrease** ▾**)** | **Result of outcome (n)**  ▴Significantly increased*  ▾Significantly decreased*  - No significant difference* | | | | | | | | |  |
| --- | --- | --- | --- | --- | --- | --- | --- | --- | --- | --- | --- |
|  |  | UCMS - adulthood  (n = 1) | | | UCMS - adolescence  (n = 1) | | | Restraint stress – adolescence (n = 1) | | | |
|  |  | ▴ | ▾ | - | ▴ | ▾ | - | ▴ | ▾ | - |  |
| **Pro-inflammatory cytokines – protein levels** |  |  |  |  |  |  |  |  |  |  |  |
| IL-1β | 1 (100%▴) | 0 | 0 | 0 | 1 | 0 | 0 | 0 | 0 | 0 |  |
| IL-6 | 2 (50%▴) | 0 | 0 | 0 | 1 | 0 | 0 | 0 | 0 | 1 |  |
| TNF-α | 3 (67%▴) | 1 | 0 | 0 | 1 | 0 | 0 | 0 | 0 | 1 |  |
| **Behavioural outcomes** |  |  |  |  |  |  |  |  |  |  |  |
| Anhedonia-like behaviour | 2 (100%▴) | 1 | 0 | 0 | 1 | 0 | 0 | 0 | 0 | 0 |  |
| Time immobile (FST) | 3 (100%▴) | 1 | 0 | 0 | 1 | 0 | 0 | 1 | 0 | 0 |  |
| Time immobile (TST) | 2 (0%▴) | 0 | 0 | 0 | 0 | 0 | 1 | 0 | 0 | 1 |  |
| Anxiety-like behaviour (OFT) | 3 (67%▴) | 1 | 0 | 0 | 0 | 0 | 1 | 1 | 0 | 0 |  |
| **Hormones/**  **metabolites** |  |  |  |  |  |  |  |  |  |  |  |
| 5-HT | 2 (50%▾) | 0 | 0 | 1 | 0 | 0 | 0 | 0 | 1 | 0 |  |
| CORT | 2 (100%▴) | 1 | 0 | 0 | 1 | 0 | 0 | 0 | 0 | 0 |  |
| DA | 2 (100%▾) | 0 | 0 | 1 | 0 | 0 | 0 | 0 | 0 | 1 |  |

Studies using adults – 25% (n = 1); studies using adolescence – 75% (n = 2).

**Abbreviations**: Behaviour: FST, forced-swim test; TST, tail-suspension test; OFT, open field test. Biological: 5-HT, serotonin; IL, interleukin; TNF, tumour necrosis factor; CORT, corticosterone*;* DA, dopamine.

* Relative to stress-free control rodents
